# Distinct brain regions mediate regulation of food intake in response to GIPR agonism or antagonism

**DOI:** 10.1038/s42255-026-01575-z

**Published:** 2026-07-24

**Authors:** Jo Edward Lewis, Mireia Montaner, Danae Nuzzaci, Paula-Peace James-Okoro, Norio Harada, Nobuya Inagaki, W. Scott Dodson, Patrick J. Knerr, Jonathan D. Douros, Fiona Mary Gribble, Frank Reimann

**Affiliations:** 1 Institute of Metabolic Science-Metabolic Research Laboratories and MRC-Metabolic Diseases Unit, University of Cambridge, Cambridge, UK; 2 Department of Endocrinology and Metabolism, School of Medical Sciences, University of Fukui, Fukui, Japan; 3 Department of Diabetes and Endocrinology, Medical Research Institute Kitano Hospital, PIIF Tazuke-Kofukai, Osaka, Japan; 4 Indiana Biosciences Research Institute, Indianapolis, IN USA

**Keywords:** Obesity, Obesity

## Abstract

The development of dual agonists for the glucagon-like peptide-1 receptor (GLP-1R) and glucose-dependent insulinotropic polypeptide receptor (GIPR) has been a landmark moment in the treatment of type 2 diabetes and obesity. However, for reasons that are incompletely understood, in preclinical and clinical studies, adding either a GIPR agonist or GIPR antagonist to GLP-1R agonism causes additional weight loss^1^. Here we show that distinct brain regions mediate the appetite-suppressing effects of GIPR agonists and the synergistic weight loss effects conferred by GIPR antagonists. We knock out *Gipr* in either the area postrema (AP) or hypothalamus of mice (Gipr^AP-KO^ and Gipr^hypo-KO^, respectively) and compare body weight and food intake responses to GIPR agonists and antagonists, alone and in combination with the GLP-1R agonist liraglutide. Gipr^AP-KO^ mice exhibit partial protection against diet-induced obesity, reduced responsiveness to the appetite-suppressing effects of acyl-GIP and a reduced ability of acyl-GIP to prevent avoidance triggered by peptide YY. Weight loss effects of liraglutide are comparable in Gipr^AP-KO^ and control mice, and the co-administration of a GIPR antagonist peptide causes similar additional weight loss in both groups. Gipr^hypo-KO^ mice, by contrast, exhibit normal appetite suppression by acyl-GIP but enhanced weight loss on liraglutide compared with control mice. Gipr^hypo-KO^ also abolishes the synergistic effect of a GIPR antagonist when combined with liraglutide—an effect that is not mediated by nucleus tractus solitarius preproglucagon neurons. GIPR antagonism and Gipr^hypo-KO^ also sensitise to cagrilintide-induced weight loss. Overall, our results suggest that the AP is responsible for the appetite-suppressing effects of GIPR agonism but that GIP receptors in the hypothalamus underlie the ability of GIPR antagonism to enhance the weight loss effects of GLP-1R and amylin receptor agonists.

## Abstract (web summary)

The study employs region-specific knockout of the glucose-dependent insulinotropic polypeptide receptor (GIPR) in either the area postrema or hypothalamus of mice to determine how GIPR agonism and antagonism regulate food intake and body weight via distinct brain regions.

## Main

Dual agonism of glucagon-like peptide-1 (GLP-1) and glucose-dependent insulinotropic polypeptide (GIP) receptors (GLP-1R and GIPR, respectively), in the form of once-weekly injectable tirzepatide, is highly effective for treating people living with obesity and type 2 diabetes, resulting in greater weight loss than GLP-1R agonism alone^2^. However, GIP biology is multimodal, and both GIPR agonism and antagonism provoke weight loss when administered together with GLP-1R agonists^3^. The results may be reconcilable by the existence of distinct *Gipr*-expressing target cell populations with different physiological roles and responsiveness. GIPR agonists decrease food intake and body weight in mice through a pathway involving *Gipr*-expressing GABAergic neurones, a population of which has been located in the area postrema (AP) of the brainstem^4–6^. Chemogenetic activation of *Gipr*-expressing neurones in either the hypothalamus or hindbrain decreases food intake in mice, suggesting more than one centrally located population of GIPR neurons regulate appetite^7,8^. Contrasting with these actions of GIPR activation to reduce food intake, *Gipr* knockout (KO) mice, and humans with impaired function GIPR variants, are protected against obesity, also probably through a central effect, as similar observations were made in global KO mice or mice lacking *Gipr* in nestin, synapsin or VGAT-expressing cells^4,5,9–12^. GIP is also anti-emetic, reducing nausea-related behaviours triggered by a variety of agents, which has been attributed to *Gipr*-expressing cells in the brainstem/AP^13–15^. By contrast, knockout of *Gipr* in GABAergic neurones did not prevent the ability of GIPR antagonists to reduce food intake^6^.

This study aimed to compare the physiological roles of GIPR in the AP and hypothalamus by targeted regional KO of *Gipr*, using a floxed *Gipr* mouse model (Gipr^fx/fx^) stereotactically injected with adeno-associated virus (AAV) encoding Cre-recombinase to generate mice with KO of *Gipr* in either the AP (Gipr^AP-KO^) or hypothalamus (Gipr^hypo-KO^). Control mice were injected with AAV encoding GFP (Gipr^AP-GFP^, Gipr^hypo-GFP^). Whereas the AP lies outside the blood–brain barrier and has a high density of *Gipr*-expressing cells, most of which are GABAergic, many *Gipr*-expressing neurones in the hypothalamus have their cell bodies behind the blood–brain barrier and include both glutamatergic and GABAergic neurons^16^.

**Fig. 1:**
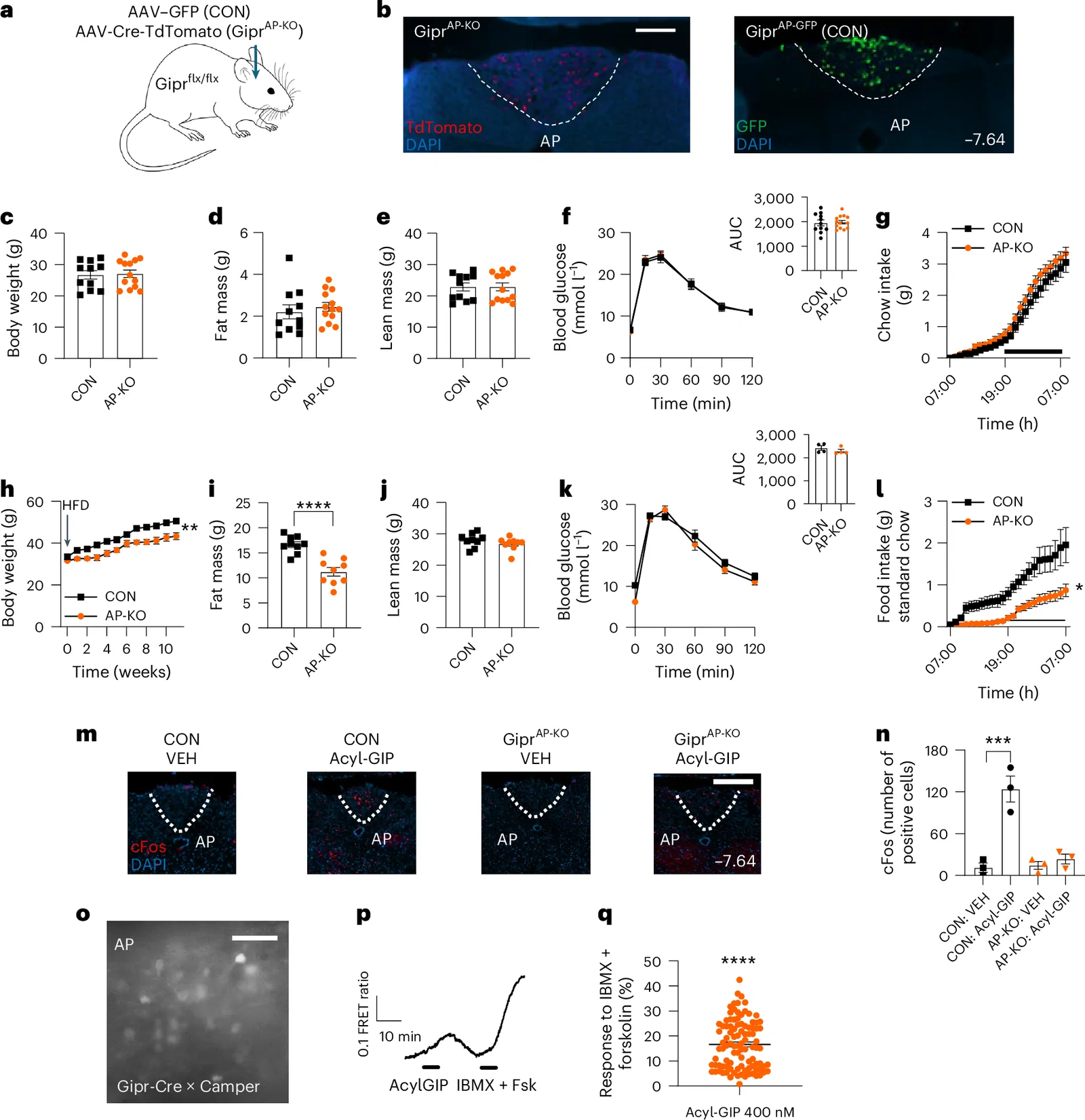
The effects of Gipr^AP-KO^ on standard chow-fed (lean) and 45% HFD fed (DIO) mice. **a**, Schematic showing the targeting of AAV–GFP (CON, Gipr^AP-GFP^) or AAV-Cre-TdTomato (Gipr^AP-KO^) to the AP of Gipr^fx/fx^ mice. **b**, Immunohistochemistry confirming targeting of AAV-Cre (example of *n* = 10) and AAV–GFP (CON) (example of *n* = 10) (scale bar, 100 µm). Additional examples are shown in Supplementary Fig. 1. **c**–**g**, Metabolic parameters of lean mice on standard chow: body weight (**c**), fat mass (**d**), lean mass (**e**), IPGTT with AUC (**f**) and food intake in metabolic cages (**g**) (CON *n* = 11, Gipr^AP-KO^ *n* = 13 per group). **h**–**l**, Metabolic parameters of DIO mice on 45% HFD: body weight (**h**), fat mass (**i**), lean mass (**j**) (*n* = 9 per group for **h**–**i**), IPGTT with AUC (*n* = 4 per group) (**k**) and HFD intake in metabolic cages (**l**) (DIO male mice, CON *n* = 5, Gipr^AP-KO^ *n* = 4). **m**,**n**, Representative cFos images of the AP of CON and Gipr^AP-KO^ animals receiving VEH or acyl-GIP (100 nmol kg^−1^; scale bar, 100 µm) (**m**) and accompanying quantification (**n**) (*n* = 3 mice per group; additional examples are shown in Supplementary Fig. 2). **o**–**q**, cAMP imaging in ex vivo brain slices: representative image of fluorescence of the AP from Gipr-Cre × CAMPER mice (scale bar, 30 µm) (**o**), representative trace of cAMP-level changes monitored as FRET ratio YFP/CFP, on perfusion with acyl-GIP and IBMX-forskolin, as indicated by the bars (**p**) and percentage FRET response to acyl-GIP relative to IBMX-forskolin treatment (*n* = 101 cells from four animals monitored in nine slices) (**q**). Data were analysed by: two-tailed Student’s (unpaired) *t*-test (**c**–**f** and **i**–**k**), Welch’s test on final weight (**h**), Mann–Whitney test on final cumulative intake (**i**), one-way ANOVA with Tukey’s multiple comparisons test (**n**) and two-tailed one-sample *t*-test (**q**). Data are mean ± s.e.m.; *P < 0.05, **P < 0.01, ***P < 0.001, ****P < 0.0001. CON, control; DAPI, 4,6-diamidino-2-phenylindole. Source data

**Extended Data Fig. 1:**
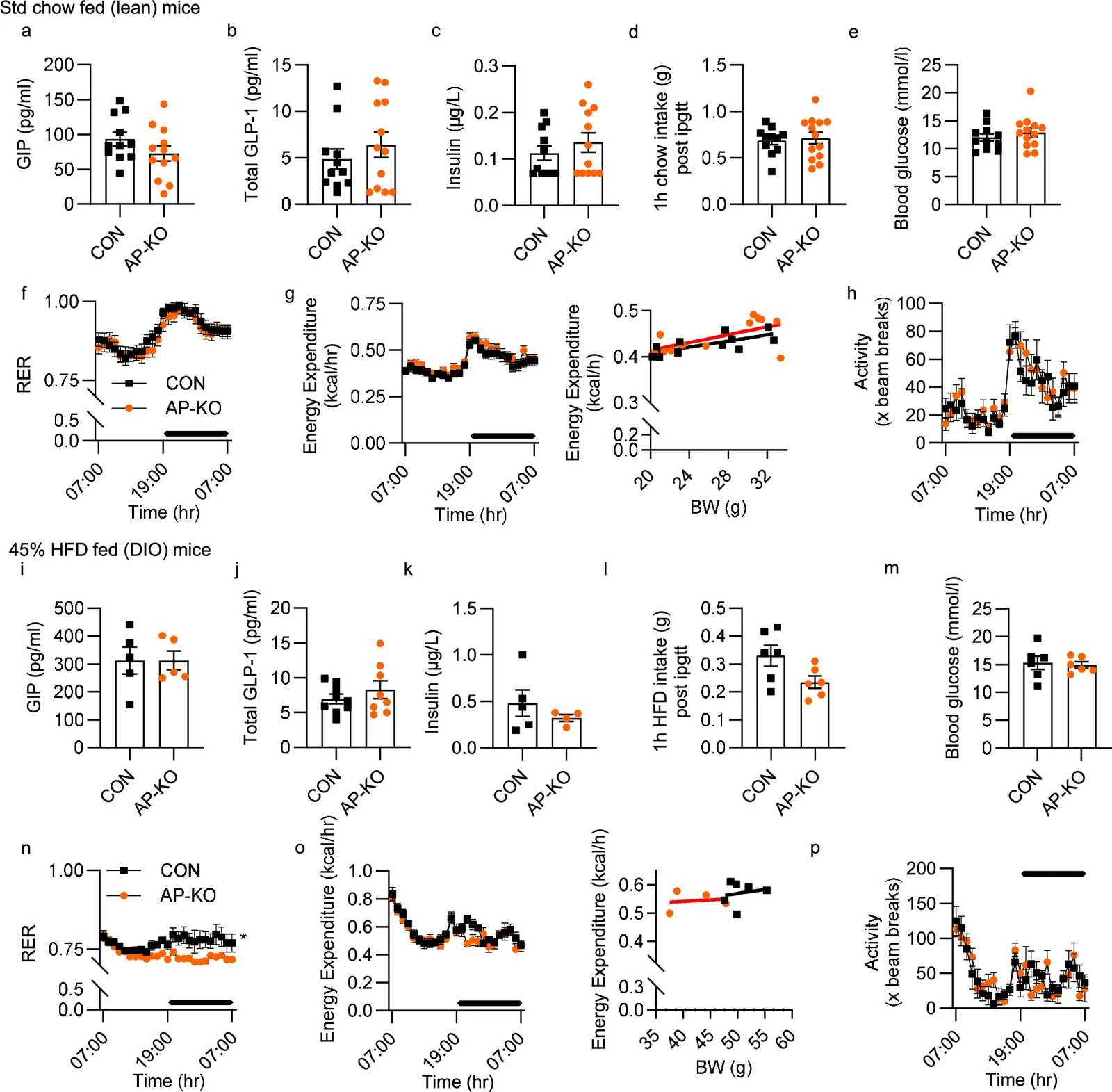
Metabolic characterisation of std chow (lean) and 45% HFD fed (DIO) Gipr^AP-KO^ mice. (**a**-**h**) Metabolic phenotype of standard chow-fed lean mice. (**a**) Fasting plasma GIP (CON n = 11, Gipr^AP-KO^ n = 12), (**b**) total GLP-1 (CON n = 11, Gipr^AP-KO^ n = 12) (**c**) insulin (CON n = 11, Gipr^AP-KO^ n = 12), (**d**) food intake post IPGTT, (**e**) ad lib fed blood glucose, (**f**) RER, (**g**) energy expenditure (inset, with ANCOVA using BW as covariate), (**h**) ambulatory activity (x beam breaks) of std chow fed (lean) mice (**d**-**h**: CON n = 11, Gipr^AP-KO^ n = 13, symbols as defined in f). (**i**-**p**) Metabolic phenotype of DIO mice fed 45% HFD. (**i**) Fasting plasma GIP (n = 5 per group), (**j**) total GLP-1 (CON n = 9, Gipr^AP-KO^ n = 8), (**k**) insulin (CON n = 5, GIPR^AP-KO^ n = 4), (l) food intake post IPGTT (CON n = 6, Gipr^AP-KO^ n = 6), (**m**) ad lib fed blood glucose (CON n = 6, Gipr^AP-KO^ n = 6), (n) RER (time x group F(24, 168) = 1.739 p = 0.023), (**o**) energy expenditure (with ANCOVA using BW as covariate), (**p**) ambulatory activity (x beam breaks). (**n**-**p**: CON n = 6, Gipr^AP-KO^ n = 4; symbols as defined in n). Data were analysed by: two-tailed Student’s unpaired t-test (**a, b, c, d, e, i, j, k, l, m**); two-way ANOVA with Sidak’s multiple comparisons test, for individual time points (f, g, h, n, o, p). Data are mean ± SEM; * p < 0.05 and *** p < 0.001. Individual *P* values are shown in the source data, unless p < 0.0001. Source data

Correct targeting of the AP in Gipr^AP-GFP^ and Gipr^AP-KO^ mice (Fig. 1a) was confirmed postmortem by immunofluorescent visualisation of TdTomato, which was encoded in the AAV-Cre virus, and GFP (Fig. 1b). Gipr^AP-KO^ mice on chow diet had comparable body weight, fat mass and lean mass to control Gipr^AP-GFP^ animals (Fig. 1c–e), mirroring the previously reported phenotypes of germline global *Gipr* KO mice and Gipr^fx/fx^ mice crossed with Nestin-Cre to drive KO throughout the central nervous system (CNS)^4,9^. Chow-fed mice with KO of *Gipr* in GABAergic neurones achieved by crossing Gipr^fx/fx^ with VGAT-Cre strains, were also previously reported to have normal body weight, although with subtle differences in fat mass and respiratory exchange ratio (RER), and improved glucose metabolism^5,11^. Gipr^AP-KO^ mice had no alterations in circulating GIP, GLP-1 or insulin (Extended Data Fig. 1a–c), intraperitoneal (i.p.) glucose tolerance (Fig. 1f, with area under the curve, AUC), food intake on refeeding after the i.p. glucose tolerance test (IPGTT) or plasma glucose during ad libitum (AL) feeding (Extended Data Fig. 1e). They also showed no differences in food intake (Fig. 1g) (compared with Gipr^AP-GFP^ mice), RER, energy expenditure or ambulatory activity, as measured in metabolic cages (Extended Data Fig. 1g,h).

Gipr^AP-KO^ mice displayed attenuated body weight gain when fed on a 45% high-fat diet (HFD), associated with reduced fat mass but preservation of lean mass (Fig. 1h–j). Fasting GIP, GLP-1 and insulin were comparable in Gipr^AP-KO^ animals and controls (Extended Data Fig. 1i–k). Glucose homeostasis as assessed by IPGTT was similar (Fig. 1k, with AUC) as was food intake following the IPGTT and AL-fed blood glucose (Extended Data Fig. 1l,m). In metabolic cages, Gipr^AP-KO^ mice showed reduced intake of HFD (Fig. 1l) and correspondingly lower RER but similar energy expenditure and ambulatory activity (Extended Data Fig. 1n–p). These characteristics are similar to those reported for GABAergic *Gipr* KO mice, which also had attenuated weight gain on HFD exposure, with reduced fat and lean mass^5,11^. Taken together, these results suggest that at least a proportion of the protection against diet-induced obesity (DIO) observed in global *Gipr* KO mice is attributable to loss of the population of *Gipr*-expressing GABAergic neurones located in the AP.

Consistent with the previously demonstrated ability of fluorescently-labelled GIP to access and activate the AP^5,7^, we found that acyl-GIP increased the total number of cells in the AP staining positive for cFos (a marker of neuronal activation) in control mice. Knockout of *Gipr* in the AP largely abolished this effect of acyl-GIP on AP cFos labelling (Fig. 1m,n). We also performed real-time imaging of cAMP levels in ex vivo hindbrain slices from Gipr-Cre × CAMPER reporter mice (Fig. 1o,p). Treatment with acyl-GIP decreased the fluorescence energy transfer ratio (FRET; YFP/CFP) in *Gipr*-labelled cells in the AP, reflecting an increase in cAMP levels (Fig. 1q). The response was 17 ± 1% (mean ± s.e.m., *n* = 101) of the maximum response to the positive control, 3-isobutyl-1-methylxanthine (IBMX) plus forskolin (Fig. 1q). These data provide high-resolution validation that acute GIP exposure directly activates (not inactivates) AP neurons.

**Extended Data Fig. 2:**
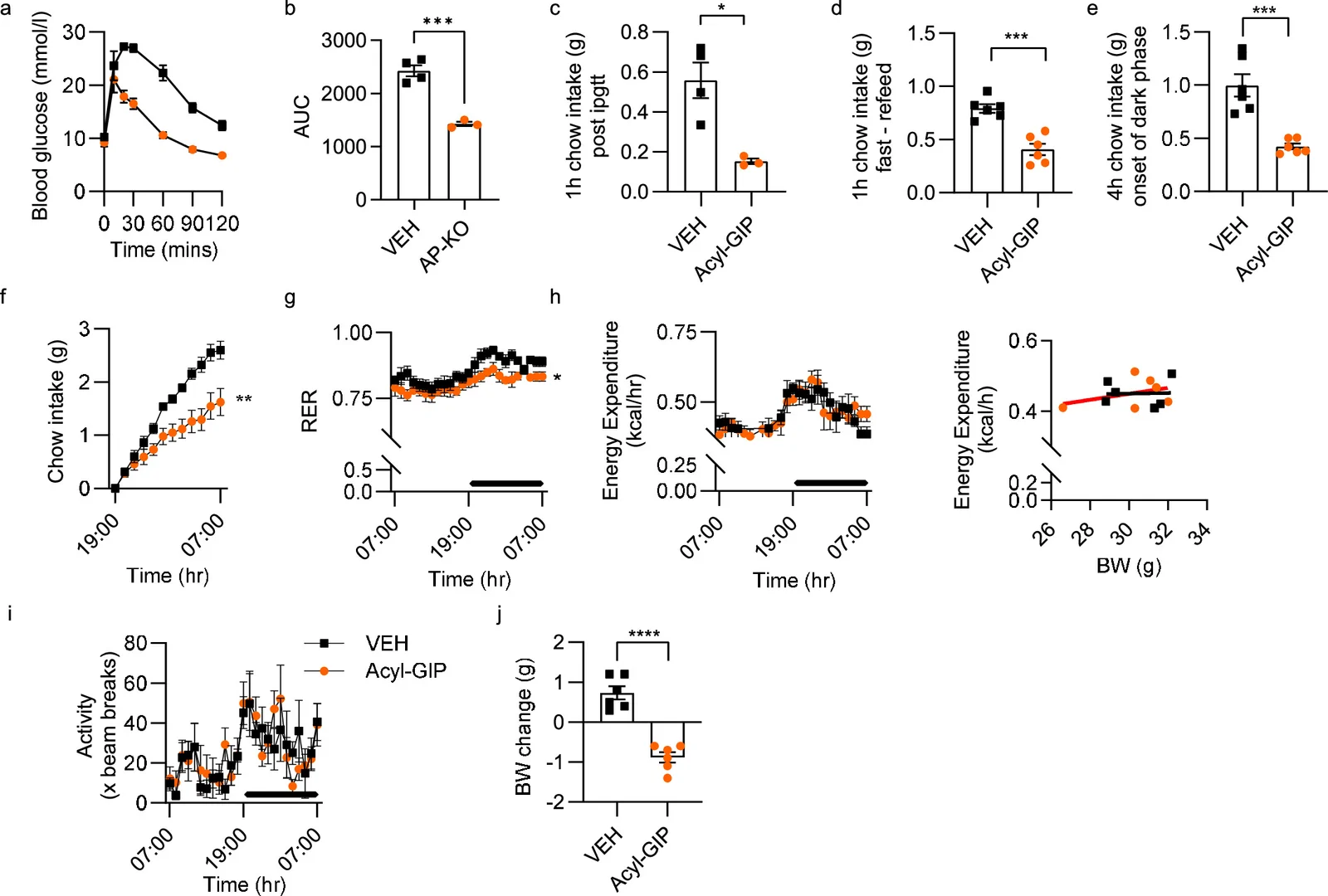
Effects of acyl-GIP on WT mice. (**a**) IPGTT with (**b**) area under the curve (AUC) and (**c**) refeeding post IPGTT of lean WT mice following treatment with vehicle (VEH; black squares) or acyl-GIP (100nmol/kg; orange circles) (**a**-**c**, VEH n = 4, acyl-GIP n = 3). Food intake (**d**) in the fast-refeed paradigm and (**e**) at the onset of the dark phase. (**f**) Food intake, (**g**) RER (effect of treatment F(1,10) = 6.34 p = 0.031), (**h**) energy expenditure (with ANCOVA using BW as covariate), (**i**) ambulatory activity and (**j**) body weight change of WT mice treated with VEH or acyl-GIP (**d**-**j**, n = 6 per group; symbols as defined in i). Data were analysed by: Student’s unpaired t-test (**b,c,d,e, j**); two-way ANOVA with Sidak’s multiple comparisons test (**g, h, i**); two-way ANOVA on final body weight with uncorrected Fisher’s LSD. Data are mean ± SEM; * p < 0.05, *** p < 0.001 and **** p < 0.0001. Individual *P* values are shown in the source data, unless p < 0.0001. Source data

Acyl-GIP improved i.p. glucose tolerance in wild type (WT) chow-fed mice (Extended Data Fig. 2a,b) and reduced subsequent food consumption (Extended Data Fig. 2c), consistent with previous observations^4,5,17^. It also reduced food intake in animals that fasted overnight and in AL-fed mice at the onset of the dark phase (Extended Data Fig. 2d,e). In a treatment-naive cohort studied in metabolic cages, administration of acyl-GIP at the onset of the dark phase decreased food intake and RER (Extended Data Fig. 2f,g) and significantly reduced body weight (Extended Data Fig. 2j). No effects were observed on energy expenditure or ambulatory activity (Extended Data Fig. 2h,i).

**Fig. 2:**
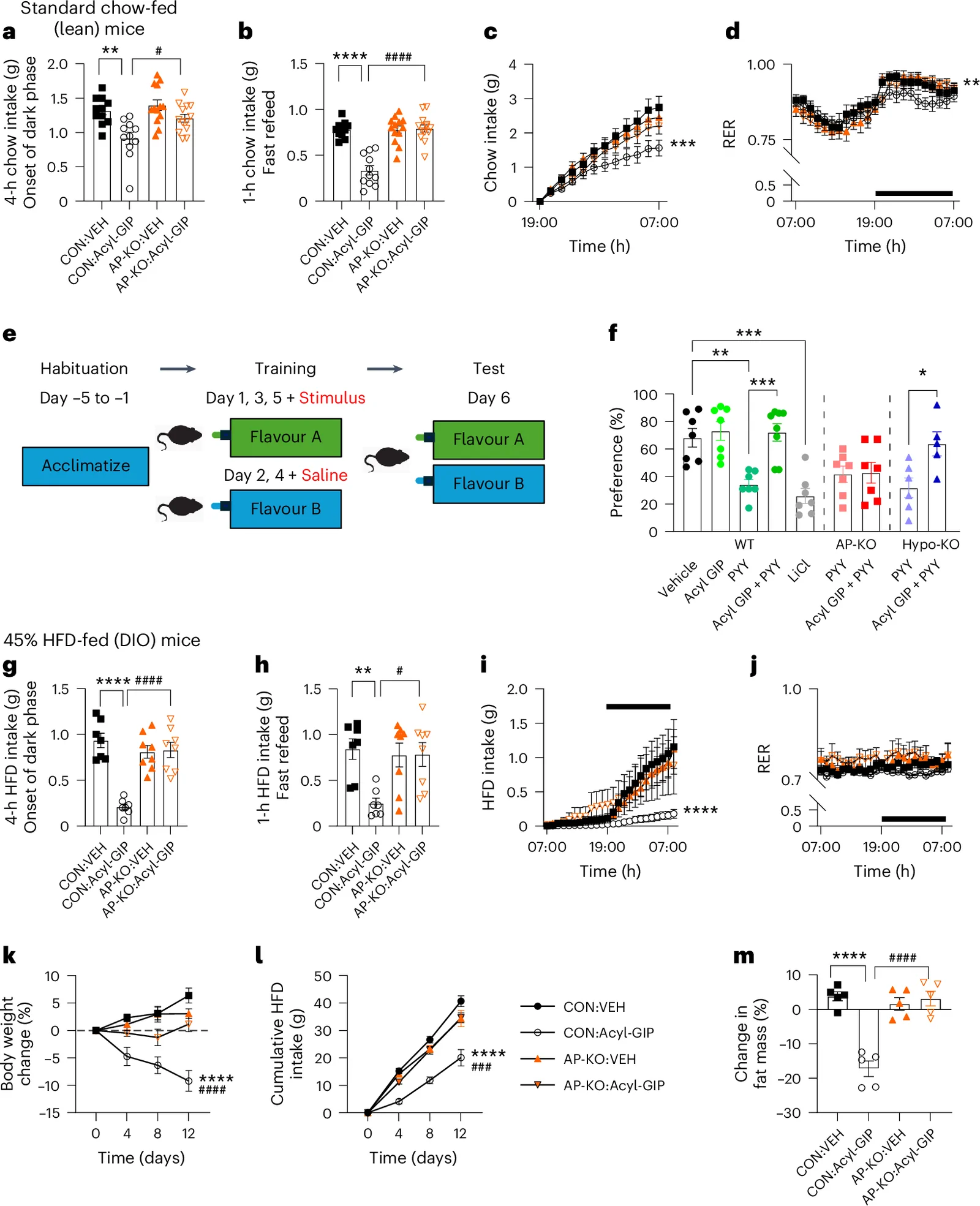
Effects of acyl-GIP on food intake in lean and DIO Gipr^AP-KO^ mice. **a**–**d**, Metabolic parameters of chow-fed lean mice: food intake at the onset of the dark phase (**a**) and in a fast-refeed paradigm (**b**) of CON (*n* = 11, Gipr^AP-GFP^) or Gipr^AP-KO^ (*n* = 13) animals receiving VEH or acyl-GIP; food intake (**c**) and RER (**d**) recorded in metabolic cages of CON (*n* = 7, Gipr^AP-GFP^) and Gipr^AP-KO^ (*n* = 8) animals receiving VEH or acyl-GIP (in **c** and **d**, *** *P* < 0.001 and **P < 0.01 comparing acyl-GIP versus VEH in CON; symbols as defined in **a** and **b**). **e**,**f**, Schematic of conditioned taste preference protocol (**e**) and observed preference for flavour (**f**) (VEH = saline, acyl-GIP and PYY at 100 nmol kg^−1^ alone or in combination or 0.2 M LiCl) in WT, Gipr^AP-KO^ or Gipr^hypo-KO^ mice (*n* = 7 per group with exception of WT/acyl-GIP + PYY (*n* = 8) and Hypo-KO (PYY *n* = 6, acyl-GIP + PYY *n* = 5). **g**,**h**, HFD intake at the onset of the dark phase (**g**) and in the fast-refeed paradigm (**h**) of CON (*n* = 7) or Gipr^AP-KO^ (*n* = 8) DIO animals receiving VEH or acyl-GIP. **i**,**j**, HFD intake (**i**) and RER (**j**) of CON or Gipr^AP-KO^ DIO animals recorded in metabolic cages, receiving VEH (*n* = 6 per group) or acyl-GIP (*n* = 4 per group; symbols as defined in **g** and **h**). **k**–**m**, Body-weight change (*n* = 6 per group, with the exception of CON/acyl-GIP (*n* = 8)) (**k**), food intake (*n* = 5 per group) (**l**) and fat mass (*n* = 5 per group) (**m**) of DIO CON or Gipr^AP-KO^ animals receiving daily VEH or acyl-GIP. ****P < 0.0001 acyl-GIP versus VEH in CON; ^###^*P* < 0.001, ^####^*P* < 0.0001 acyl-GIP in CON versus Gipr^AP-KO^; symbols as defined in **l** and **m**. Data were analysed by: one-way ANOVA with Tukey’s multiple comparisons test (**a**, **b**, **g**, **h** and **m**) or Sidak’s multiple comparisons test (**f**); two-way ANOVA including time-series data (**d** and **j**), two-way ANOVA on final food intake or body weight with uncorrected Fisher’s least significant difference (LSD) (**c**, **i**, **k** and **l**). Data are mean ± s.e.m.; *P < 0.05, **P < 0.01, ***P < 0.001 and ****P < 0.0001. Source data

**Extended Data Fig. 3:**
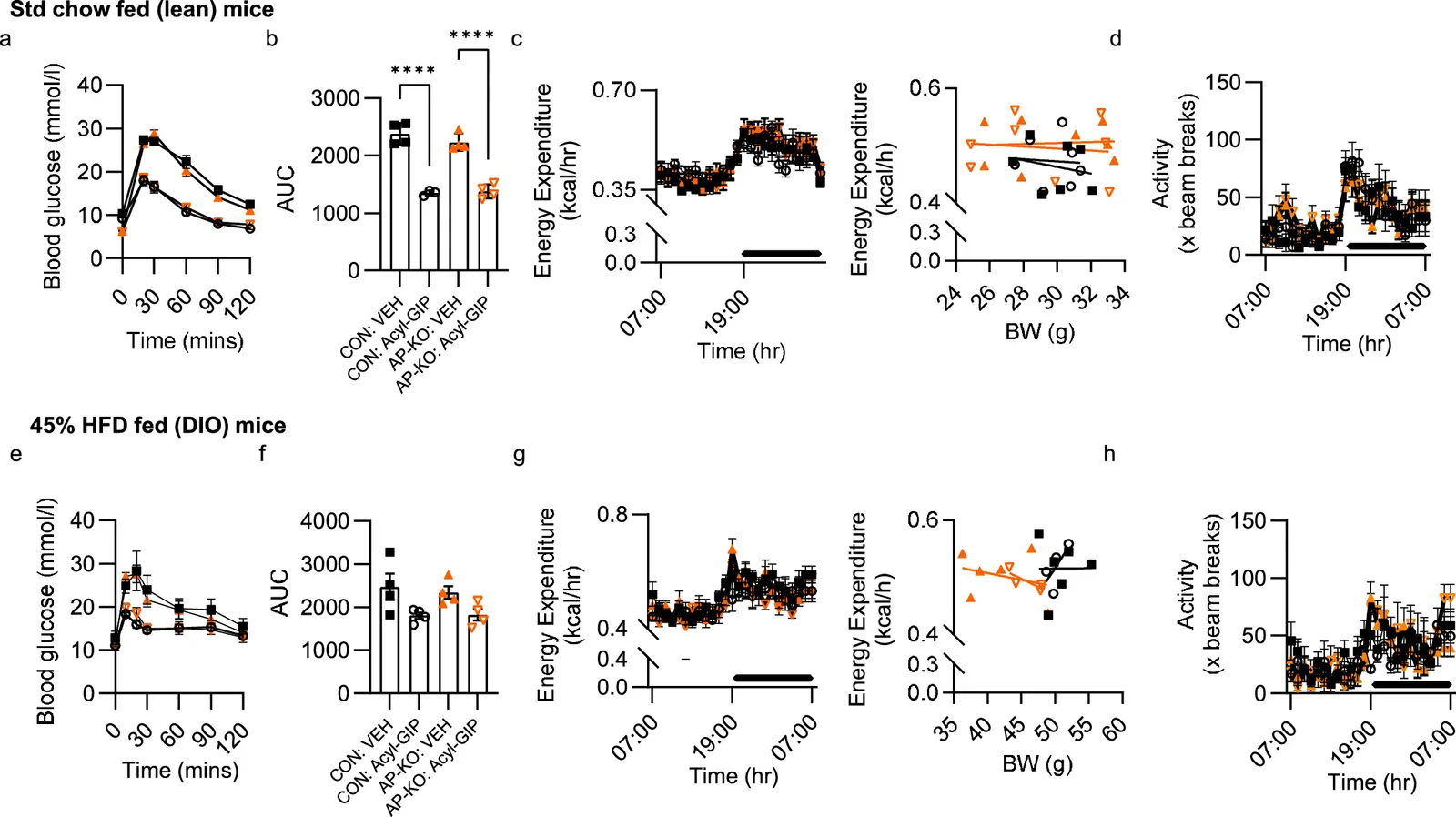
Effects of acyl-GIP in lean and DIO Gipr^AP-KO^ mice. (**a**-**d**) Metabolic phenotype of chow-fed CON (Gipr^AP-GFP^) and Gipr^AP-KO^ mice treated with VEH or acyl-GIP (100nmol/kg). (**a**) IPGTT with (**b**) AUC (CON VEH n = 4, CON acyl-GIP n = 3, Gipr^AP-KO^ VEH n = 4 and Gipr^AP-KO^ n = 4). (**c**) Energy expenditure recorded in metabolic cages (with ANCOVA using BW as covariate) and (**d**) corresponding ambulatory activity (**c**,**d**: CON VEH n = 7, CON acyl-GIP n = 7, Gipr^AP-KO^ n = 8 and Gipr^AP-KO^ acyl GIP n = 8). (**e**-**h**) Metabolic phenotype of DIO mice on 45% HFD and treated with VEH or acyl-GIP. l/kg). (**e**) IPGTT with (**f**) AUC (n = 4 per group). (**g**) Energy expenditure recorded in metabolic cages (with ANCOVA using BW as covariate) and (**h**) corresponding ambulatory activity (**g**,**h**: CON VEH n = 6, CON acyl-GIP n = 4, Gipr^AP-KO^ n = 6, Gipr^AP-KO^ n = 4). Symbols throughout as defined in b/f. Data were analysed by: one-way ANOVA with Tukey’s multiple comparisons test (**b**, **f**); two-way ANOVA with Tukey’s multiple comparisons test (**a**, **c**, **d**, **e**, **g**, **h**). Data are mean ± SEM; **** p < 0.0001. Individual *P* values are shown in the source data, unless p < 0.0001. Source data

While previous studies have shown that CNS GABAergic neurons are necessary for the food intake- and body weight-reducing effects of GIPR agonists^5,11^, the anatomic region that underlies these effects remains unknown. KO of *Gipr* in the AP (Gipr^AP-KO^) abolished the ability of acyl-GIP to reduce food intake at the onset of the dark phase in AL-fed animals, using Gipr^AP-GFP^ mice as a control group (Fig. 2a). Similar results were apparent in a fast-refeed paradigm—treatment with acyl-GIP significantly reduced food intake in control Gipr^AP-GFP^ animals highly motivated to feed after a 16-h fast, while this effect was lost in Gipr^AP-KO^ animals (Fig. 2b). The effects of acyl-GIP on i.p. glucose tolerance were maintained in Gipr^AP-KO^ mice as expected, consistent with direct effects of acyl-GIP on pancreatic β-cells rather than an indirect effect mediated via the AP (Extended Data Fig. 3a,b). In metabolic cages, acyl-GIP reduced food intake across the 12-h dark period, associated with a significant reduction in the RER in control Gipr^AP-GFP^ but not Gipr^AP-KO^ mice (Fig. 2c,d). No differences were observed in energy expenditure or activity (Extended Data Fig. 3c,d).

**Fig. 3:**
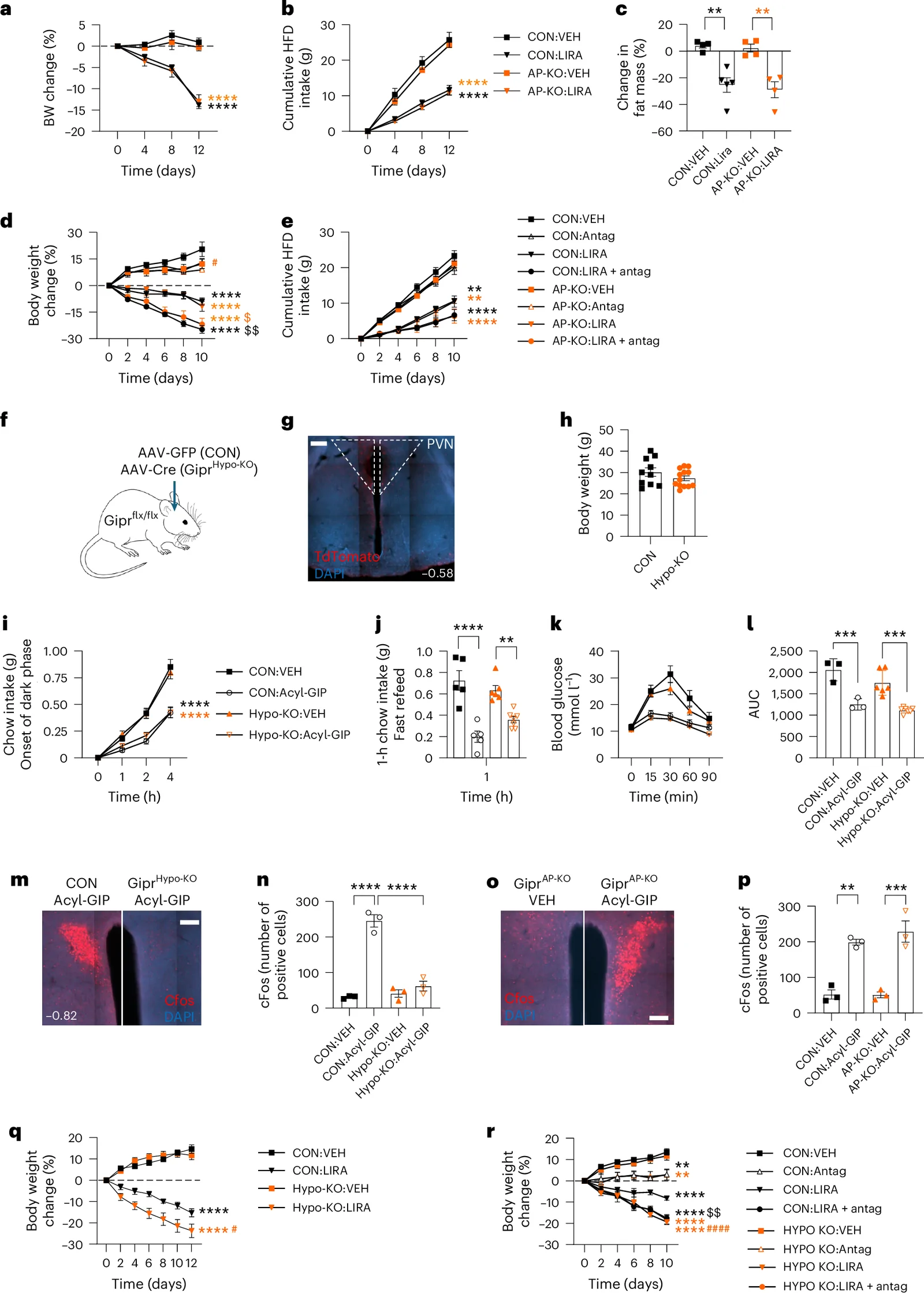
Effects of GLP-1R agonism and GIPR antagonism on Gipr^AP-KO^ and Gipr^Hypo-KO^ mice. **a**–**c**, GLP-1RA responsiveness of DIO Gipr^AP-KO^ mice: body weight change (**a**), cumulative food intake (**b**) and delta fat mass (**c**) of CON (Gipr^AP-GFP^) and Gipr^AP-KO^ mice receiving VEH or LIRA daily (100 nmol kg^−1^ (*n* = 4, except CON LIRA *n* = 5 per group; symbols as defined in **b** and **c**); black, **P < 0.01, ****P < 0.0001 LIRA versus VEH in CON; orange, **P < 0.01, ****P < 0.0001 LIRA versus VEH in GIPR^AP-KO^. **d**,**e**, Body weight change (**d**) and cumulative HFD intake (**e**) of CON and Gipr^AP-KO^ mice transferred to a 45% HFD and treated as indicated (CON VEH *n* = 5, Gipr^AP-KO^ VEH *n* = 5, CON antag *n* = 6, Gipr^AP-KO^ antag *n* = 6, CON LIRA *n* = 5, Gipr^AP-KO^ LIRA *n* = 5, CON LIRA + antag *n* = 5 and Gipr^AP-KO^ *n* = 7; symbols as defined in **e**); black, **P < 0.01, ****P < 0.0001 treatment versus VEH in CON; orange, **P < 0.01, ****P < 0.0001 treatment versus VEH in Gipr^AP-KO^; black, ^$$^*P* < 0.01 versus CON LIRA; orange, ^$^*P* < 0.05 versus AP-KO LIRA; orange, ^#^*P* < 0.05 antag in CON versus AP-KO. **f**–**q**, Phenotype of Gipr^hypo-KO^ mice. Schematic showing the targeting of AAV–GFP (CON) or AAV-Cre (Gipr^hypo-KO^) to the hypothalamus of Gipr^fx/fx^ mice (**f**). Immunohistochemistry confirming targeting of AAV-Cre (which co-expresses TdTomato, example of *n* = 10, with additional examples shown in Supplementary Fig. 3; *n* = 10 for AAV-Cre and *n* = 8 (two missing owing to brain processing issues) for AAV–GFP control) (**g**). Body weight (CON *n* = 10, Gipr^hypo-KO^ *n* = 13) (**h**), chow intake at the onset of the dark phase (CON *n* = 10, Gipr^hypo-KO^ *n* = 13; black ****P < 0.0001 versus VEH in CON; orange, ****P < 0.0001 versus VEH in Gipr^hypo-KO^) (**i**), chow intake in the fast-refeed paradigm of CON or Gipr^hypo-KO^ mice receiving VEH or acyl-GIP (100 nmol kg^−1^, CON VEH/acyl-GIP *n* = 5, Gipr^hypo-KO^ VEH *n* = 6, Gipr^hypo-KO^ acyl-GIP *n* = 7; symbols as defined in **i**) (**j**). IPGTT (**k**) with AUC of CON and Gipr^hypo-KO^ mice receiving VEH or acyl-GIP (CON VEH *n* = 3, CON acyl-GIP *n* = 3, Gipr^hypo-KO^ VEH *n* = 6, Gipr^hypo-KO^ acyl-GIP *n* = 7) (**l**). cFos in the PVN of CON and Gipr^hypo-KO^ animals receiving acyl-GIP (**m**), which is quantified (**n**) (*n* = 3 animals per group; additional examples are shown in Supplementary Fig. 4). cFos in the PVN of Gipr^AP-KO^ mice treated with VEH or acyl-GIP (**o**), which is quantified (**p**) (*n* = 3 animals per group; additional examples are shown in Supplementary Fig. 4). Body weight change of CON and Gipr^Hypo-KO^ mice transferred to a 45% HFD and treated with VEH or LIRA as indicated (*n* = 6 per group; black, ****P < 0.0001 versus VEH in CON; orange, ****P < 0.0001 versus VEH in Gipr^hypo-KO^; orange, ^#^*P* < 0.05 LIRA in CON versus Gipr^hypo-KO^) (**q**). **r**, The same as in **q** with the addition of GIPR-antag (co)treatment (CON VEH *n* = 5, Gipr^hypo-KO^ *n* = 6, CON antag *n* = 5, Gipr^hypo-KO^ antag *n* = 6, CON LIRA *n* = 7, Gipr^hypo-KO^ LIRA *n* = 7, CON LIRA + antag *n* = 6, Gipr^hypo-KO^ LIRA + antag *n* = 7; black, **P < 0.01, ****P < 0.0001 versus VEH in CON; orange, **P < 0.01, ****P < 0.0001 versus VEH in Gipr^hypo-KO^; ^####^*P* < 0.0001 LIRA in CON versus Gipr^hypo-KO^; ^$$^LIRA versus LIRA + antag in CON). Data were analysed by: two-way ANOVA on final food intake or body weight with uncorrected Fisher’s LSD (**a**, **b**, **d**, **e**, **i**, **q**and **r**); one-way ANOVA with Tukey’s multiple comparisons test (**c**); and one-way ANOVA with Dunnett’s test for multiple comparisons (**h**, **j**, **l**, **n** and **p**). Data are mean ± s.e.m.; *P < 0.05, **P < 0.01, ***P < 0.001 and ****P < 0.0001. Source data

Brainstem *Gipr*-expressing cells have been implicated in suppressing nausea-related behaviours, such as conditioned taste avoidance (CTA), as long-acting GIPR agonists reduced CTA triggered by PYY(3-36), GLP-1 or GDF15 administration^13–15,18,19^. To determine whether GIPR in the AP is responsible for attenuating the avoidance effect of PYY, we applied a CTA assay in which exposure to PYY was paired with a novel tastant alone or in combination with acyl-GIP. In this experimental design, reduced consumption of the paired stimulus is indicative of an avoidance response (Fig. 2e). In line with previous reports, conditioning with PYY, but not acyl-GIP or vehicle (VEH) control, decreased preference for the paired taste in lean mice. Acyl-GIP abolished the avoidance effect of PYY in control GIPR^AP-GFP^ animals but not in GIPR^AP-KO^ animals (Fig. 2f), confirming the importance of AP *Gipr*-expressing cells for mediating this action of GIPR agonism. By contrast, knockout of *Gipr* in the hypothalamus (generated as shown in Fig. 3f) did not prevent this anti-aversive effect of acyl-GIP.

Gipr^AP-KO^ and Gipr^AP-GFP^ mice were next fed an HFD to induce DIO. At the onset of the dark phase, acyl-GIP significantly reduced food intake of DIO Gipr^AP-GFP^ but not Gipr^AP-KO^ animals (Fig. 2g), and similar results were apparent in the 16-h fast-refeed paradigm (Fig. 2h). The effects of acyl-GIP on i.p. glucose tolerance were maintained in both groups (Extended Data Fig. 3e,f). In metabolic cages, acyl-GIP reduced food intake and RER across the 12-h dark period in DIO Gipr^AP-GFP^ but not Gipr^AP-KO^ mice (Fig. 2i,j), with no differences in energy expenditure or activity (Extended Data Fig. 3g,h). Chronic daily treatment with acyl-GIP reduced body weight and food intake in control DIO Gipr^AP-GFP^ mice, associated with a significant reduction in fat mass. These effects were absent in Gipr^AP-KO^ animals treated with acyl-GIP (Fig. 2k–m). The results indicate that *Gipr*-expressing cells in the AP underlie the ability of GIPR agonists to reduce food intake in lean and DIO mice and, taken together with the previous report that GABAergic *Gipr* KO mice do not exhibit reduced food intake in response to acyl-GIP, suggest that subpopulations of GABAergic *Gipr*-expressing neurones located in the AP mediate both the appetite-suppressing and anti-aversive effects of exogenous GIPR agonists^6^.

There is emerging evidence that GIPR agonism and antagonism exert their paradoxically similar effects on weight loss via distinct neuronal populations^6,12^. We sought to further delineate these divergent regulatory networks by investigating whether AP GIPRs modulate responses to either GLP-1R agonism or the combination of GLP-1R agonism with GIPR antagonism. In a first study, DIO Gipr^AP-GFP^ and Gipr^AP-KO^ mice received daily injections of the GLP-1R agonist liraglutide (LIRA; 100 nmol kg^−1^). Over 12 days of treatment, LIRA reduced body weight, food intake and fat mass, similarly in Gipr^AP-KO^ and control Gipr^AP-GFP^ mice, compared with VEH control-injected groups (Fig. 3a–c). This indicated that the lack of *Gipr* in the AP did not in itself sensitize to GLP-1R agonism. In a subsequent study, lean Gipr^AP-KO^ and Gipr^AP-GFP^ mice were switched to an HFD on the same day as initiating 10 days of daily injections with either LIRA (or VEH) alone, or LIRA (or VEH) plus GIPR antagonist peptide (GIPR-antag)^6^. LIRA + GIPR-antag induced additional weight loss and food intake suppression compared with LIRA alone; however, there was no difference between Gipr^AP-KO^ and control Gipr^AP-GFP^ groups (Fig. 3d,e). Thus, although GIPR in the AP is required for the weight loss effects of GIPR agonists, it is not the target of therapeutic GIPR antagonists, supporting recent findings that *Gipr* KO in GABAergic neurons also abolished responses to GIPR agonism but not antagonism^6^. Nevertheless, the target for GIPR antagonists is believed to be a neuronal population in the brain, as a peptide–antibody conjugate that blocks GIPR while activating GLP-1R required central GIPR for maximal weight loss in DIO mice, as determined using mice lacking *Gipr* in cells expressing synapsin^12^.

The results mirror a previous observation from our group, that hypothalamic *Gipr* KO appeared to sensitize DIO mice to weight loss induced by a long-acting GLP-1R agonist—a finding that had not been the focus of that study and remained unexplained at the time but has since also been observed in global GIPR-KO mice^7,11^. We therefore generated new cohorts of mice lacking *Gipr* in the hypothalamus (Gipr^hypo-KO^), by targeted injection of AAV-Cre or control AAV–GFP (Gipr^hypo-GFP^) into the paraventricular nucleus (PVN) (Fig. 3f). Targeting was confirmed by the localised expression of TdTomato from the AAV-Cre virus (Fig. 3g). Gipr^hypo-KO^ mice displayed normal body weight on a chow diet (Fig. 3h). The ability of acyl-GIP to reduce food intake at the onset of the dark phase and in the 16-h fast-refeed paradigm was maintained in Gipr^hypo-KO^ mice (Fig. 3i,j), as was the effect of acyl-GIP on glucose tolerance (Fig. 3k,l). Treatment with acyl-GIP increased cFos staining in the PVN of control Gipr^hypo-GFP^ but not GIPR^hypo-KO^ animals (Fig. 3m,n), suggesting that GIP receptors located in the hypothalamus underlie the observed hypothalamic cFos activation triggered by peripheral GIP. By contrast, KO of *Gipr* in the AP did not prevent cFos staining in the PVN (Fig. 3o,p).

Gipr^hypo-KO^ and control Gipr^hypo-GFP^ mice were transferred to an HFD and treated daily with either LIRA or VEH. LIRA reduced body weight in both groups, but the magnitude of the weight loss was greater in the Gipr^hypo-KO^ cohort (Fig. 3q), suggesting that these animals are sensitised to GLP-1R agonism, supporting our previous observation that sensitivity to GLP-1R agonism is enhanced in mice lacking *Gipr* in the hypothalamus^7^. We next repeated this experimental paradigm in Gipr^hypo-KO^ and Gipr^hypo-GFP^ mice, combining the initiation of HFD with a combination of LIRA (or VEH) alone or LIRA (or VEH) plus GIPR antagonist peptide (GIPR-antag). As before, LIRA caused greater weight loss in Gipr^hypo-KO^ than in Gipr^hypo-GFP^ mice, and LIRA + GIPR-antag caused more weight loss than LIRA alone in the control Gipr^hypo-GFP^ group (Fig. 3r). However, the addition of GIPR-antag to LIRA did not further enhance weight loss in the Gipr^hypo-KO^ mice, supporting the idea that the GIP receptors required for weight loss effects of GIPR antagonism are located in the hypothalamus.

**Fig. 4:**
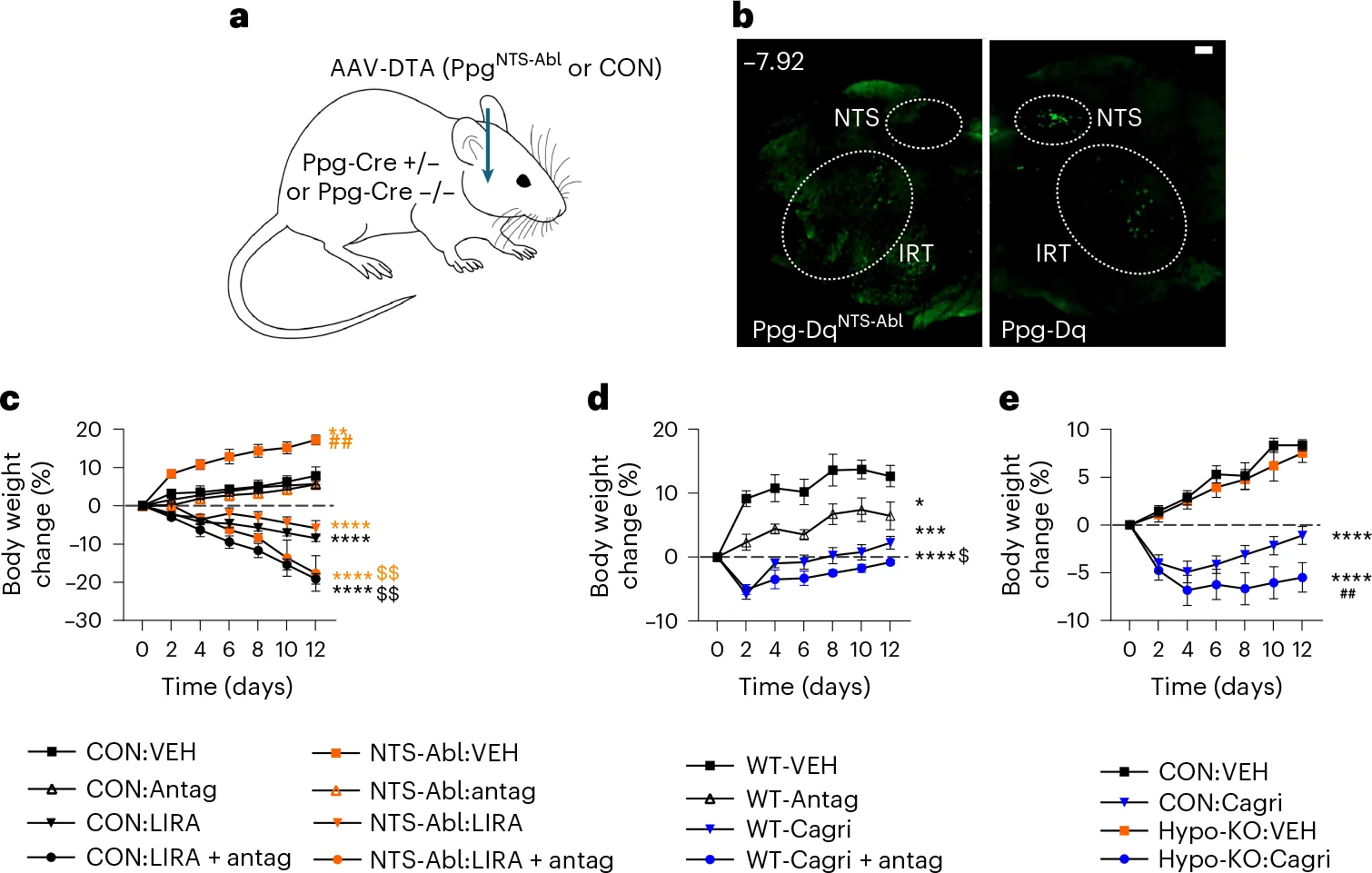
Interactions of GIPR antagonism with brainstem Ppg neurons and amylin receptors. **a**, Schematic showing generation of Ppg-neuron ablation in the NTS by bilateral stereotactic injection of AAV-DTA into the NTS of Ppg-Cre-positive (Ppg^NTS-Abl^) or Ppg-Cre-negative (CON) mice. **b**, Immunohistochemistry confirming ablation of the Ppg population in the NTS (scale bar, 100 µm, example of *n* = 6; additional examples shown in Supplementary Fig. 5); green fluorescence of Cre-reporters (Dq-DREADD/NuTRAP-labelling Ppg-Cre neurons) is still observed in the intermediate reticular nucleus (IRT) but not the NTS in AAV-DTA-injected animals (Ppg-Dq^NTS-Abl^), contrasting with control Ppg-Cre animals also carrying a Dq-reporter (Ppg-Dq) shown on the right, which show GFP labelled cells in both the NTS and the IRT. **c**, Body weight change of CON and Ppg-Dq^NTS-Abl^ mice transferred to a 45% HFD and treated with VEH, LIRA or LIRA + GIPR-antag as indicated (CON VEH *n* = 6, CON antag *n* = 5, CON LIRA *n* = 6, CON LIRA + antag *n* = 7; Ppg^NTS-Abl^ VEH *n* = 5, Ppg^NTS-Abl^ antag *n* = 5, Ppg^NTS-Abl^ LIRA *n* = 6 and Ppg^NTS-Abl^ LIRA + antag *n* = 6); black, ****P < 0.0001 versus VEH in CON; orange, **P < 0.01, ****P < 0.0001 versus VEH in Ppg^NTS-Abl^; orange, ^##^*P* < 0.01 VEH in CON versus Ppg^NTS-Abl^; black, ^$$^*P* < 0.01 versus LIRA in CON; orange, ^$$^*P* < 0.01 versus LIRA in Ppg^NTS-Abl^ mice. **d**, Body weight change of WT mice transferred to a 45% HFD and treated daily with VEH, Cagri (30 nmol kg^−1^), GIPR-antag or Cagri + antag, as indicated (VEH *n* = 8, Cagri *n* = 8, antag *n* = 8, cagri + antag *n* = 7). *P < 0.05, ***P < 0.001, ****P < 0.0001 versus VEH; ^$^*P* < 0.05 Cagri versus Cagri + antag by two-tailed unpaired *t*-test. **e**, Body weight change of CON or Gipr^hypo-KO^ mice transferred to a 45% HFD and treated daily with VEH or Cagri (30 nmol kg^−1^) (CON VEH *n* = 5, Gipr^hypo-KO^ VEH *n* = 5, CON Cagri *n* = 10, Gipr^hypo-KO^ Cagri *n* = 8). Data are mean ± s.e.m.; ****P < 0.0001 versus CON VEH, ^##^*P* < 0.01 Cagri in CON versus GiprR^hypo-KO^. Data were analysed by: two-way ANOVA on final body weight with uncorrected Fisher’s LSD (**c**–**e**). Source data

Previously, treatment with a GIPR-antag was found to result in a hindbrain transcriptomic signature similar to GLP-1R agonism^6^. In the CNS, GLP-1 is produced by preproglucagon (Ppg) neurons with most cell bodies found in the nucleus tractus solitarius (NTS)^20^. We hypothesised that the reported brainstem transcriptomic changes might reflect modulation of NTS Ppg neuron activity downstream of GIPR antagonism in the hypothalamus and therefore tested the effect of GIPR-antag on mice with ablated NTS Ppg neurons (Ppg^NTS-Abl^) generated by bilateral stereotactic injection of AAV-DTA into the NTS of Ppg-Cre or control mice (Cre-negative mice receiving AAV-DTA into the NTS) (Fig. 4a,b). Groups were treated with VEH, GIPR-antag, LIRA and LIRA + GIPR-antag. Whereas Ppg^NTS-Abl^ increased body weight gain on transfer to an HFD, and GIPR-antag attenuated this effect, ablation of the Ppg neuronal population did not attenuate the effects of any treatment arm (Fig. 4c).

Cagrilintide (Cagri), a long-acting amylin analogue that targets amylin receptors in the hindbrain, reduces food intake and body weight in mice^21^. To determine whether the weight loss sensitization effect of GIPR-antag extended to Cagri, we transferred WT mice to an HFD and treated with VEH, Cagri, GIPR-antag and the combination (Cagri + antag). Treatment with GIPR-antag reduced body weight gain, while Cagri transiently reduced body weight. The combination resulted in greater weight loss (Fig. 4d). To determine whether hypothalamic *Gipr*-expressing cells underlie this effect, Gipr^hypo-KO^ mice were treated with VEH or Cagri. In this cohort, Cagri induced a greater but still transient weight loss in WT animals, while in Gipr^hypo-KO^ mice, even greater weight loss was sustained to the end of the experiment (Fig. 4e).

Taken together, our results indicate that GIPR in the AP underlie the weight loss effects of GIPR agonism, whereas GIPR in the hypothalamus underlie the additional weight loss effects of adding GIPR antagonism to GLP-1R or amylin receptor agonism. Despite years of confusion around the relative benefits of GIPR agonism versus antagonism, the clinical picture that both can enhance the weight-lowering efficacy of a GLP-1R agonist seems clear. Both GIPR co-agonism (as in tirzepatide) and GIPR antagonism (as in MariTide, a bispecific hybrid molecule comprising two GLP-1R agonist peptides conjugated to a monoclonal anti-GIPR antagonistic antibody) are effective in treating people living with obesity^2,3,22^.

Previously, we have shown that chemogenetic activation of GIPR-expressing cells in either the hypothalamus or the hindbrain supresses food intake^7,8^, and GIPR-expression in oligodendrocytes has recently been linked to enhanced access of co-administered GLP-1R agonists^23^. We now demonstrate that GIPR agonist administration critically relies on *Gipr* expression in the hindbrain, while GIPR antagonism relies on hypothalamic *Gipr* expression—both are probably neuronal populations, given that we used hSyn-driven Cre-expression for the region-specific knockout studies. While it may be argued that our study used relatively small group sizes, the results were significant and reproducible, consistent with the large effect sizes and low variability of our experimental paradigms. The finding of distinct central populations of *Gipr*-expressing neurones in the AP and hypothalamus underlying responses to agonism and antagonism, respectively, goes some way towards resolving the dilemma of agonism and antagonism having similar outcomes.

Our work suggests that GIPR antagonism does not require the central production of GLP-1, as ablation of the Ppg neuron population had little effect on the weight loss outcomes. The observation that GIPR antagonism also sensitized to the non-incretin-based weight loss drug Cagri opens potential new avenues for therapeutic exploration. Future work is still, however, required to identify the neuronal networks underlying GIPR interactions in the AP and hypothalamus and their crosstalk with other appetite-regulating circuitry.

## Methods

### Animal studies

All experiments were performed under the UK Home Office project licence PE50F6065 in accordance with the UK Animals (Scientific Procedures) Act, 1986, and approved by the University of Cambridge Animal Welfare and Ethical Review Body. All mice were from a C57BL/6 background and were group-housed and maintained in individual ventilated cages with standard bedding and enrichment in a temperature- and humidity-controlled room on a 12-h light:dark cycle (lights on 7:00) with AL access to food (standard diet, Scientific Animal Food Engineering, or 45% HFD) and water unless otherwise stated. DIO mice received 45% HFD for at least 12 weeks before the study. GIPR^fx/fx^ mice were as described in ref. ^24^. GIPR-Cre × CAMPER mice were previously described^8^. Ppg mice were as described in ref. ^25^.

### Surgery

Mice, under isoflurane anaesthesia and receiving Metacam before surgery, were stereotactically implanted with a temporary guide cannula (Plastics One) positioned above the AP (anterior/posterior (A/P), 0.5 mm; dorsal/ventral (D/V), 3 mm; and lateral, 0.0 mm from the optical crest), NTS (A/P, 0.5 mm; D/V, 3 mm; and lateral, ±0.4 mm from the optical crest) or PVN (A/P, −1.1 mm; D/V, 4.9 mm; and lateral, −0.3 mm from the bregma). The cannula-holding bar was placed in a 10^0^ rostro-caudal angle for AP injections (adapted from ref. ^26^). Bevelled stainless-steel injectors (33 gauge, Plastics One) extending 2 mm (for AP and NTS) and 1 mm (from PVN) from the tip of the guide were used for injections. A total of 35 and 100 nl AAV-hSyn-Cre-p2a-dTomato (Addgene, 107738-AAV9), AAV-hEF1α-dlox-DTA (AAV-DTA as per ref. ^27^) or AAV-hSyn-GFP (as control; Addgene, 50465-AAV9) was injected (diluted to 1 × 10^12^ vg ml^−1^) to the AP, NTS and PVN, respectively. Mice were allowed a minimum of 2 weeks recovery before testing. Studies were performed in male and female mice. Chronic HFD DIO studies were limited to male mice, as female mice display attenuated weight gain^28,29^.

### Body composition

Fat and lean masses were calculated by a quantitative magnetic resonance system (EchoMRI-100H, Medical Systems).

### Peptide drug treatment

Mice were treated via subcutaneous (s.c.) injection at the indicated doses with either long-acting acyl-GIP (100 nmol kg^−1^ s.c.), LIRA (100 nmol kg^−1^ s.c.) or an acylated peptide GIPR antagonist ([Nα-Ac,L14,R18,E21]hGIP(5–31)-K11(γE-C16)) (1500 nmol kg^−1^ s.c.). Cagri (Cambridge Bioscience) was administered at 30 nmol kg^−1^ s.c., as per ref. ^21^. Structures, representative synthetic procedures, in vitro validation of receptor activity and in vivo validation of weight-lowering efficacy in DIO mice for all peptide drugs have been published previously^30,31^.

### Glucose tolerance via IPGTT

Mice were fasted overnight (<16 h). At the zero timepoint, glucose was administered at 2 g kg^−1^ body weight i.p. and either VEH or acyl-GIP was delivered i.p. contralaterally. Blood glucose was measured via the tail vein at just before (‘0’) and 15, 30, 60, 90 and 120 min after administration (~5 µl, Accu-Chek). A minimum duration of 72 h between testing was used. Diet was then presented for 1 h with intake measured (fast refeed after IPGTT), and an AL-fed blood glucose measurement was taken.

### Food intake

Acute food intake studies were performed in a cross-over design, on age-matched groups, a minimum of 72 h apart (unless otherwise stated) at the onset of the dark phase (19:00). Animals were singly housed before the experiment and fasted for 2 h. Mice were administered VEH or acyl-GIP (as previous). Food intake was measured at the timepoints indicated. For the fast-refeed experiment, animals were fasted overnight (<16 h) before presentation of the diet for 1 h (during the light phase).

### Metabolic cages

Animals were singly housed (for 5 days) and acclimatised to metabolic cages before study and data collection. Oxygen consumption and carbon dioxide production were determined using an indirect calorimetry system (Promethion, Sable Systems). The system consisted of 16 metabolic cages (similar to home cages), equipped with water bottles and food hoppers connected to load cells for continuous monitoring, in a temperature- and humidity-controlled room. The RER and energy expenditure (via the Weir equation) were calculated, while ambulatory activity was determined simultaneously. Raw data were processed using ExpeData (Sable Systems). Animals were exposed to standard chow or an HFD during metabolic assessment (as indicated). Animals were treated with VEH or acyl-GIP at the onset of the dark phase (19:00).

### Plasma hormone measurement

All blood samples were collected by capillary EDTA tubes via the tail vein in free-moving, conscious animals. Blood samples were placed immediately on ice, plasma collected after centrifugation and stored at −80 °C until required. Circulating hormones were measured using enzyme-linked immunosorbent assay (ELISA; MesoScale Discovery, insulin and total GLP-1) at Core Biochemical Assays Laboratories. GIP was measured using ELISA as per the manufacturer’s instructions (Millipore).

### Immunohistochemistry

Tissues were collected as previously described^27^. In brief, mice were perfused 90 min after treatment. Tissue slices were stained overnight with primary antisera (Supplementary Table 1) before incubation with appropriate secondary antisera (Supplementary Table 1). Slides were imaged using an Axioscan Z1 slide scanner (Zeiss) and confocal microscope (Leica TCS SP8 X). For cFos studies, two to six sections per mouse at the level of the hypothalamus (bregma −0.46 to −1.06 mm) and hindbrain (bregma −7.2 to −7.76 mm) were counted bilaterally and averaged across sections for each mouse. Images were analysed in ImageJ.

### Live brain slice imaging

Acute hindbrain slices were prepared as previously^27^. Mice were anaesthetized using sodium pentobarbital at a dose of 180 mg kg^−1^ (Dolethal, Vetoquinone). The animals were intracardially perfused with oxygenated high-Mg^2+^/low-Ca^2+^ ice-cold artificial cerebrospinal fluid (perfusion solution; composition in mM: 2.5 KCl, 200 sucrose, 28 NaHCO_3_, 1.25 NaH_2_PO_4_, 8 glucose, 7 MgCl_2_, 0.5 CaCl_2_; pH 7.4). The brain was dissected and extracted from the skull and immersed in oxygenated perfusion solution. Subsequently, 250-µm-thick slices of the hindbrain containing the AP were cut on a Vibratome (Leica) and immersed in a recovery solution (in mM: 3 KCl, 118 NaCl, 25 NaHCO_3_, 1.2 NaH_2_PO_4_, 2.5 Glucose, 7 MgCl_2_, 0.5 CaCl_2_; pH 7.4) at 34 °C for 30 min. Slices were then transferred to standard artificial cerebrospinal fluid (aCSF; in mM: 3 KCl, 118 NaCl, 25 NaHCO_3_, 10 glucose, 1MgCl_2_, CaCl_2_; pH 7.4) at room temperature for 1 h before recording. All solutions were continuously bubbled with 95% O_2_/5% CO_2_.

Imaging was performed using a fixed-stage upright microscope (Olympus, BX51W1). Slices were anchored in a glass-bottom chamber using a harp (Warner instruments) and imaged using a 40× water immersion objective. GIPR-Camper neurons of the AP were excited using a light-emitting diode (LED) light source (Dual Optoled, Cairn Research) at 435 nm for 50 ms every 3 s, and CFP and YFP emissions were simultaneously recorded after passing through an optosplit (Cairn Research; 465–505 and 525–600 nm, respectively). Images were captured using a camera (Evolve 512 Delta, Photometrics), acquisitions were saved and data were logged using Metamorph software (Molecular Devices). Slices were continuously perfused with room temperature standard aCSF at a flow rate of 1 ml min^−1^. Acyl-GIP (400 nM), IBMX (100 µM, I7018, Sigma) and forskolin (10 µM, F6886, Sigma) were added directly in standard aCSF enriched with 0.3% casein (Sigma).

Identifiable cell bodies and an area determined for background fluorescence were outlined for each image and the mean pixel intensity extracted for each region of interest (ROI). The mean intensity of the background was subtracted from each ROI. The FRET of the mTurquoise FRET donor (CFP) and Venus FRET acceptor (YFP) was calculated. Acyl-GIP responses were measured by calculating the maximum change in FRET on adding acyl-GIP, as a percentage of the maximum change in FRET on adding IMBX-forskolin for each cell.

### CTA

For CTA, mice were housed in cages that permitted AL access to water from two bottles side by side for at least 5 days before training and experimentation. On days 1, 3 and 5 of the study, animals were water deprived overnight before presentation of two bottles containing the same novel flavoured solution (either cherry or grape, plus sucralose). Following 40 min of access, mice were treated as indicated (acyl-GIP at 100 nmol kg^−1^, PYY at 100 nmol kg^−1^ (Bachem 4018880), acyl-GIP + PYY or LiCl at 0.2 M (Sigma 7447-41-8) or VEH (PBS containing 0.05% Tween80)). Mice were replaced and had continued access to the solutions for a further 40 min. On training days 2 and 4, mice received the alternative flavour paired with VEH treatment. On day 6, mice were presented with two bottles containing the flavours. Consumption was measured for a 5-h period. CTA was quantified using

$$\begin{array}{l}{\text{Choice index (conditioned flavour}})\\={{\text{Volume consumed}}}_{{\text{conditioned flavour}}}/({{\text{Volume consumed}}}_{{\text{alternative flavour}}}\\\,+{{\text{Volume consumed}}}_{{\text{conditioned flavour}}})\end{array}$$

### Statistics

Data were plotted using GraphPad Prism 10.6.1 software. Statistical analysis was performed by *t*-test (unpaired or paired, as indicated), one-way analysis of variance (ANOVA), two-way ANOVA (time × treatment or genotype × treatment) with post hoc comparisons (where appropriate, as indicated in the figure legends). *N* represents biological replicates. Sample size was computed on the basis of pilot data and previously published data. Data are presented as mean ± s.e.m. and probabilities of *P* < 0.05 were considered statistically significant in all tests.

### Reporting summary

Further information on research design is available in the Nature Portfolio Reporting Summary linked to this article.

## Supplementary information

Supplementary Information Supplementary Figs. 1–5 and Table 1.

Reporting Summary

Peer Review File

Supplementary Data 1 The tif files for Supplementary Figs. 1–5, for example, uncropped tif for all images shown, including the ones reproduced partly cropped in Figs. 1–4.

## Source data

Source Data Fig. 1 Raw and statistical source data.

Source Data Fig. 2 Raw and statistical source data.

Source Data Fig. 3 Raw and statistical source data.

Source Data Fig. 4 Raw and statistical source data.

Source Data Extended Data Fig. 1 Raw and statistical source data.

Source Data Extended Data Fig. 2 Raw and statistical source data.

Source Data Extended Data Fig. 3 Raw and statistical source data.

## Data Availability

All data used for the statistical analysis are available in the Source data, along with the GraphPad Prism-derived report on the statistical analysis. The statistical report contains the mean difference between the groups, the significance summary and the exact *P* values, where appropriate (unless *P* < 0.0001). Source data are provided with this paper.
