# Supplementary material for: Distinct brain regions mediate regulation of food intake in response to GIPR agonism or antagonism
Source: Nat Metab. 2026 Jul 24;8(8):1669–78. doi: 10.1038/s42255-026-01575-z (PMC13489937; doi:10.1038/s42255-026-01575-z)
Supplement: Supplementary file 1 [file 42255_2026_1575_MOESM1_ESM.pdf]

nature metabolism

Supplementary information https://doi.org/10.1038/s42255-026-01575-z

Distinct brain regions mediate regulation of food intake in response to GIPR agonism or antagonism

In the format provided by the authors and unedited

Supplementary figure 1: Targeting confirmation for AP injections

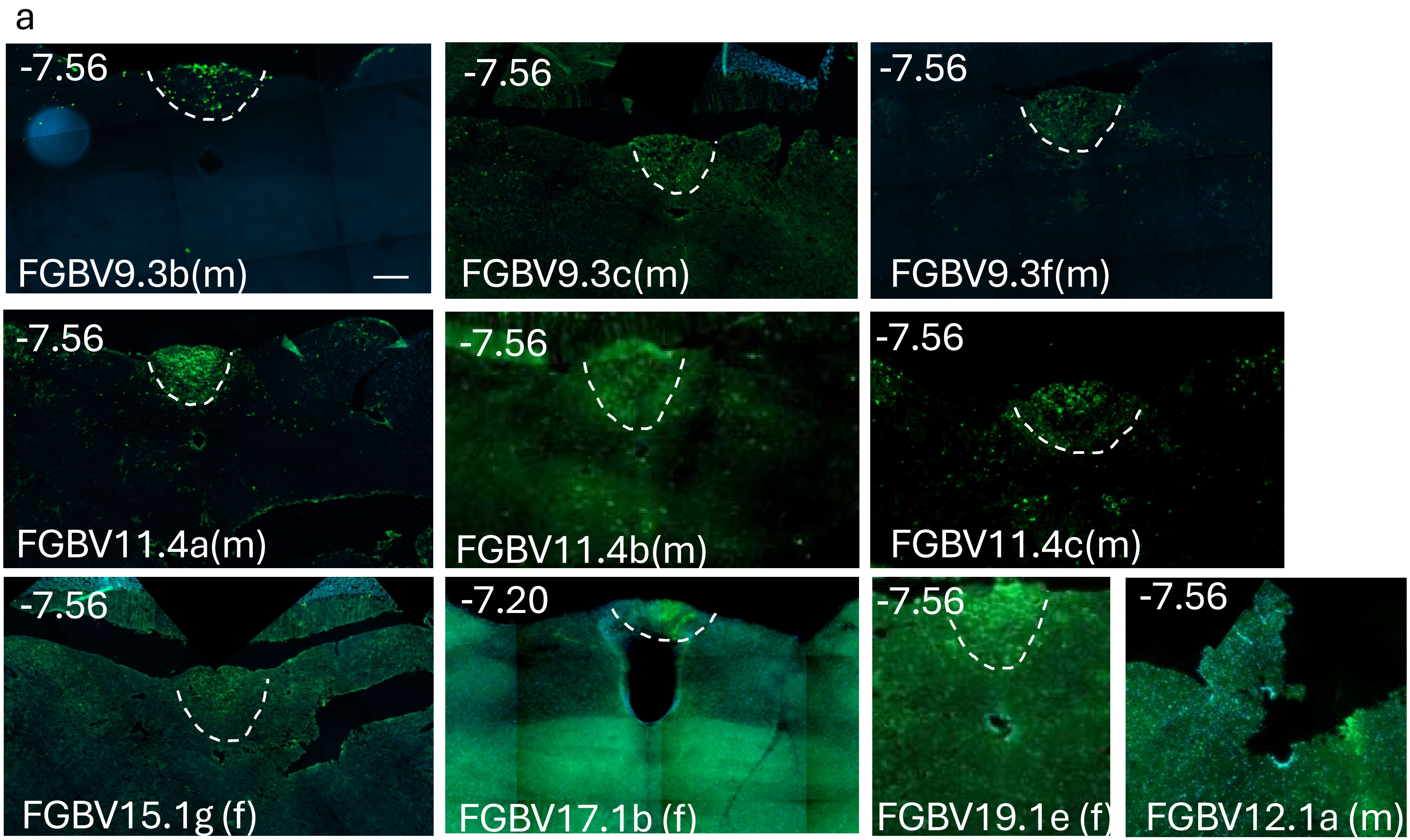

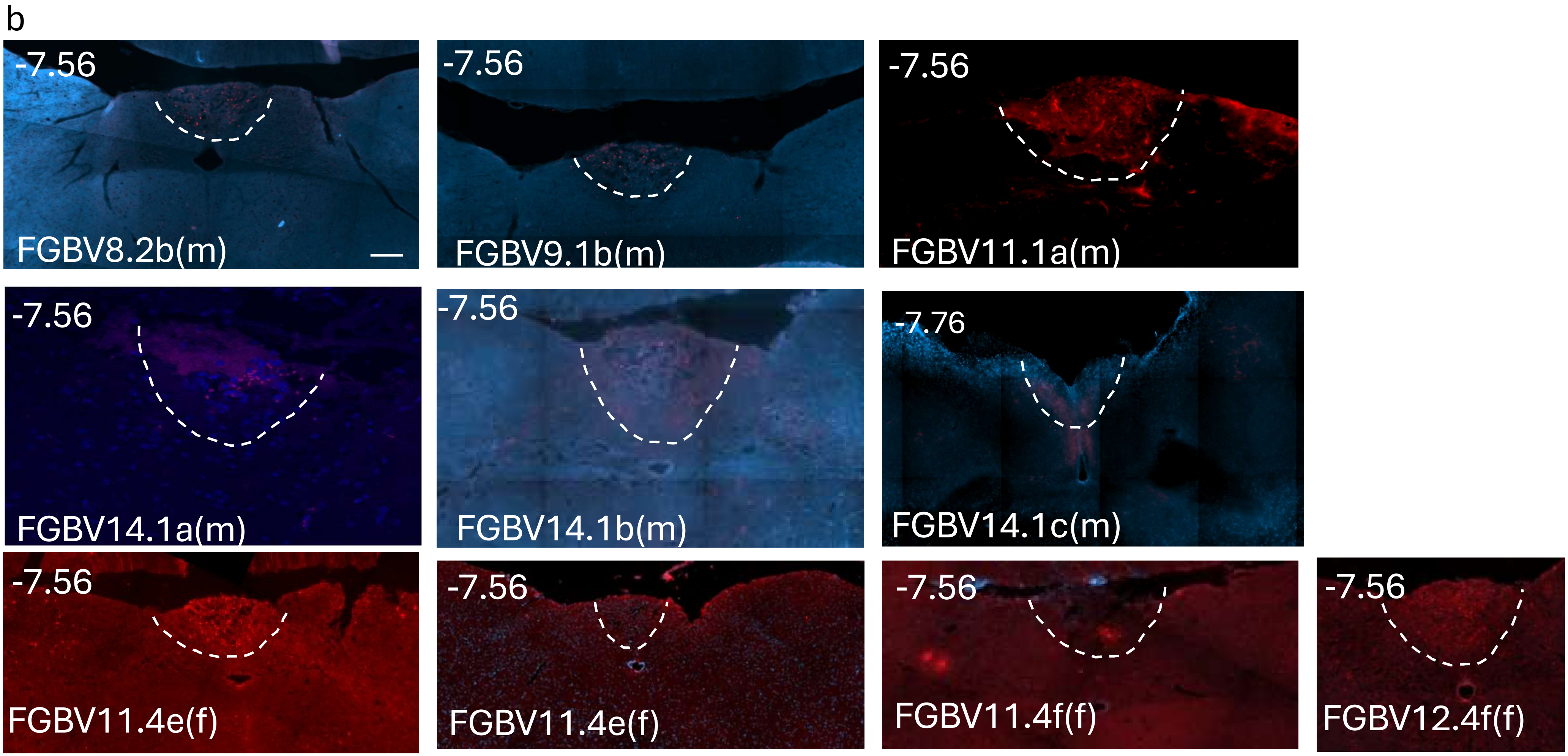

Supplementary figure 1: Targeting of the AP injections. Targeting of a) AAV-GFP (CON) and b) AAV-Cre-TdTomato (Gipr$^{AP\text{-}KO}$) to the AP (as outlined) of GIPR$^{fx/fx}$ mice. FGBV denotes colony and animal ID (sex m/f as indicated). Bregma as specified, scale bar = 100μm.

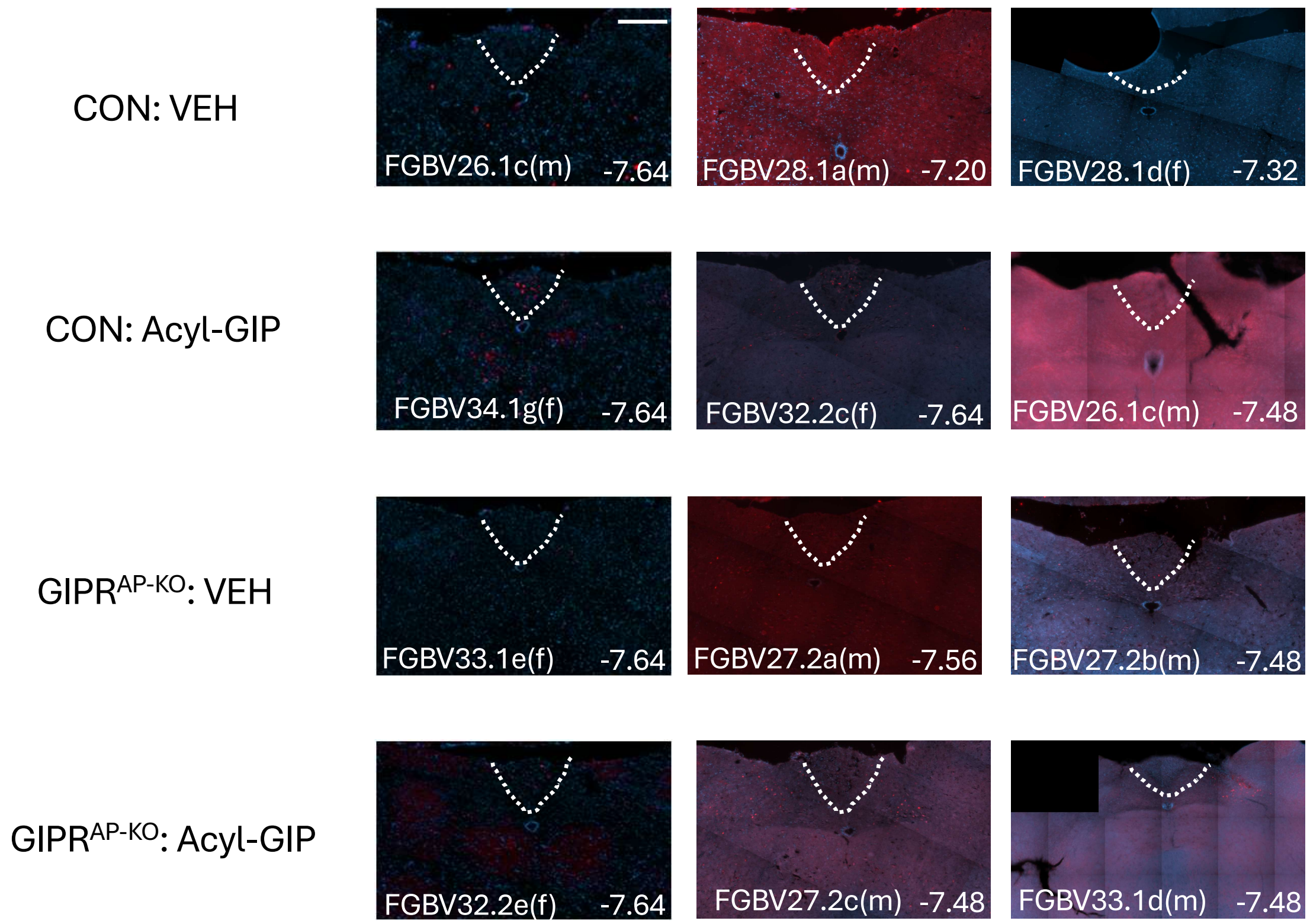

Supplementary figure 2: cFos labelling in the AP. Representative cfos images of the AP (outlined, n = 2-6 sections per mouse) of CON or Gipr^{AP-KO} mice receiving VEH or acyl-GIP (as indicated). FGBV denotes colony and animal ID (sex m/f as indicated). Bregma as specified, scale bar = 100µm.

Supplementary figure 3: Targeting confirmation for hypothalamic injections

a

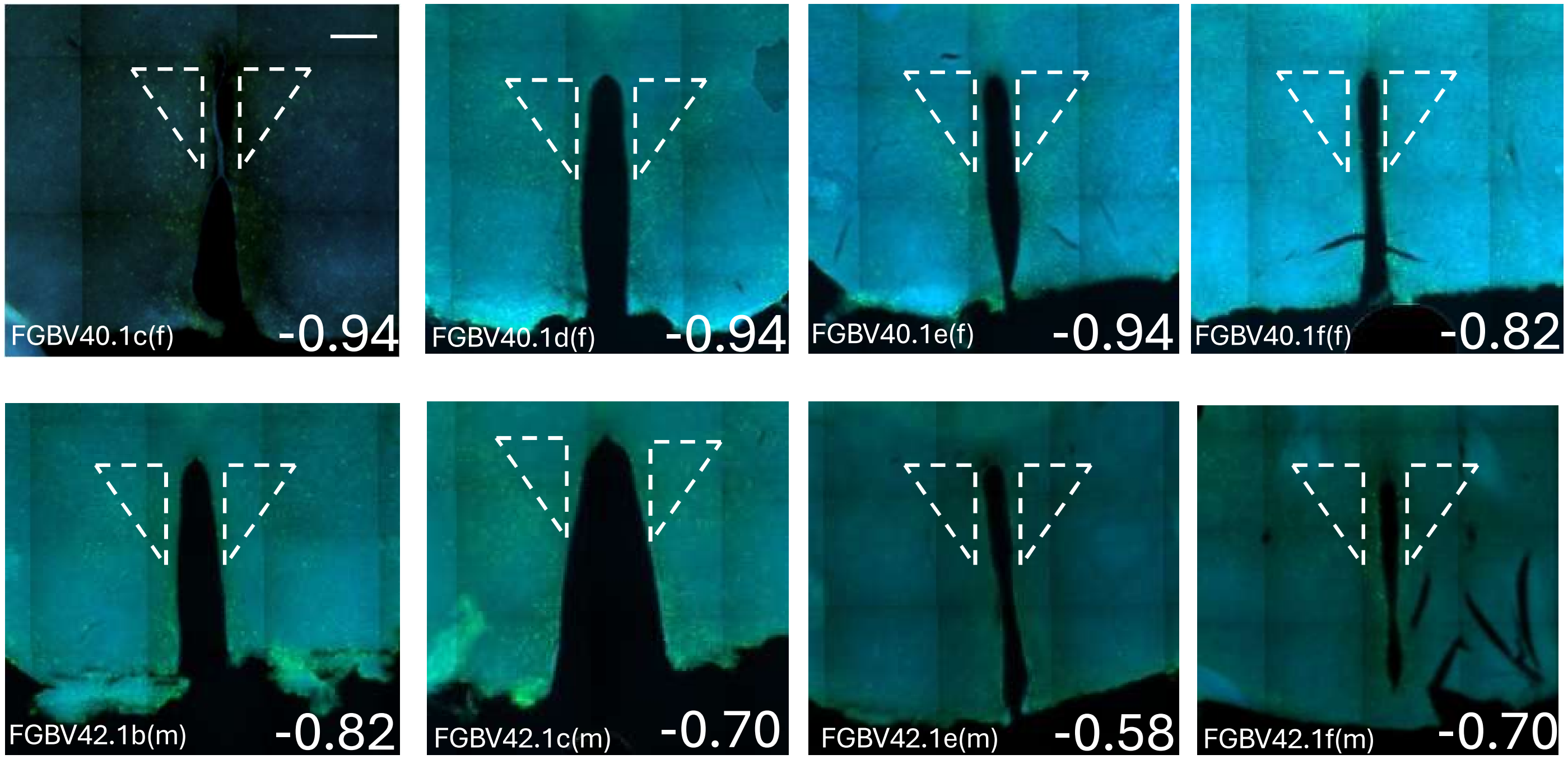

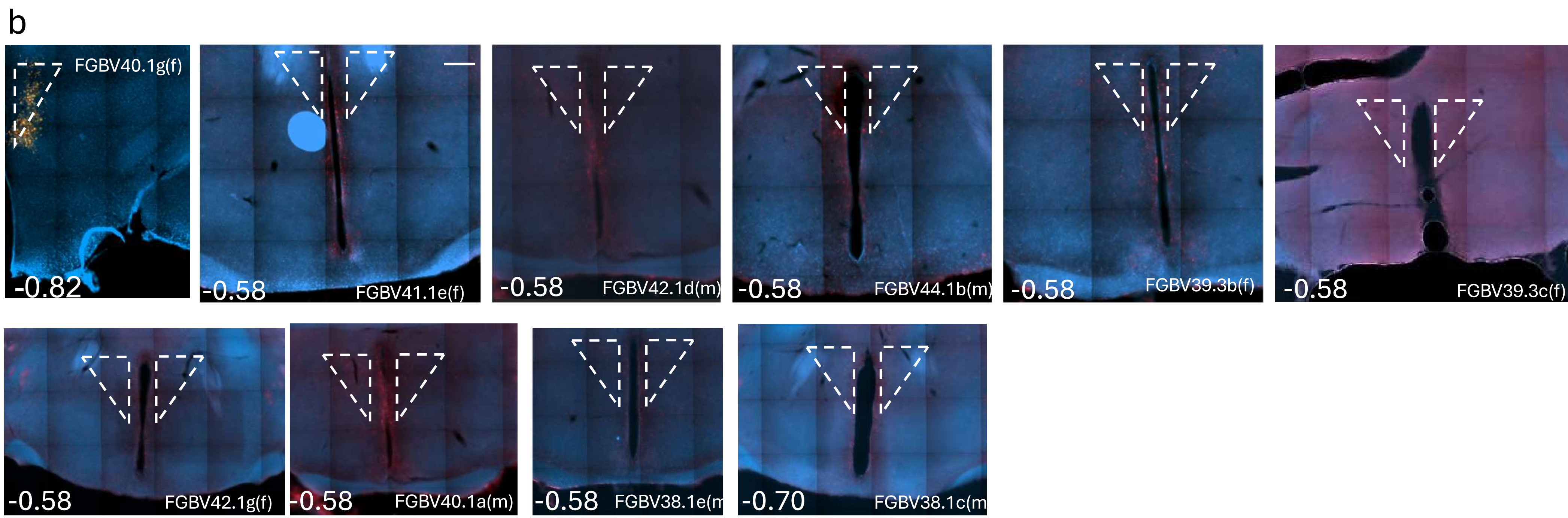

Supplementary figure 3: Targeting confirmation for hypothalamic injections. Targeting of a) AAV-GFP (CON) and b) AAV-Cre-TdTomato (Gipr$^{hypo\text{-}KO}$) to the hypothalamus (PVN outlined) of GIPRfx/fx mice. FGBV denotes colony and animal ID (sex m/f as indicated). Bregma as specified, scale bar = 100µm.

Supplementary figure 4: cFos labelling in the hypothalamus

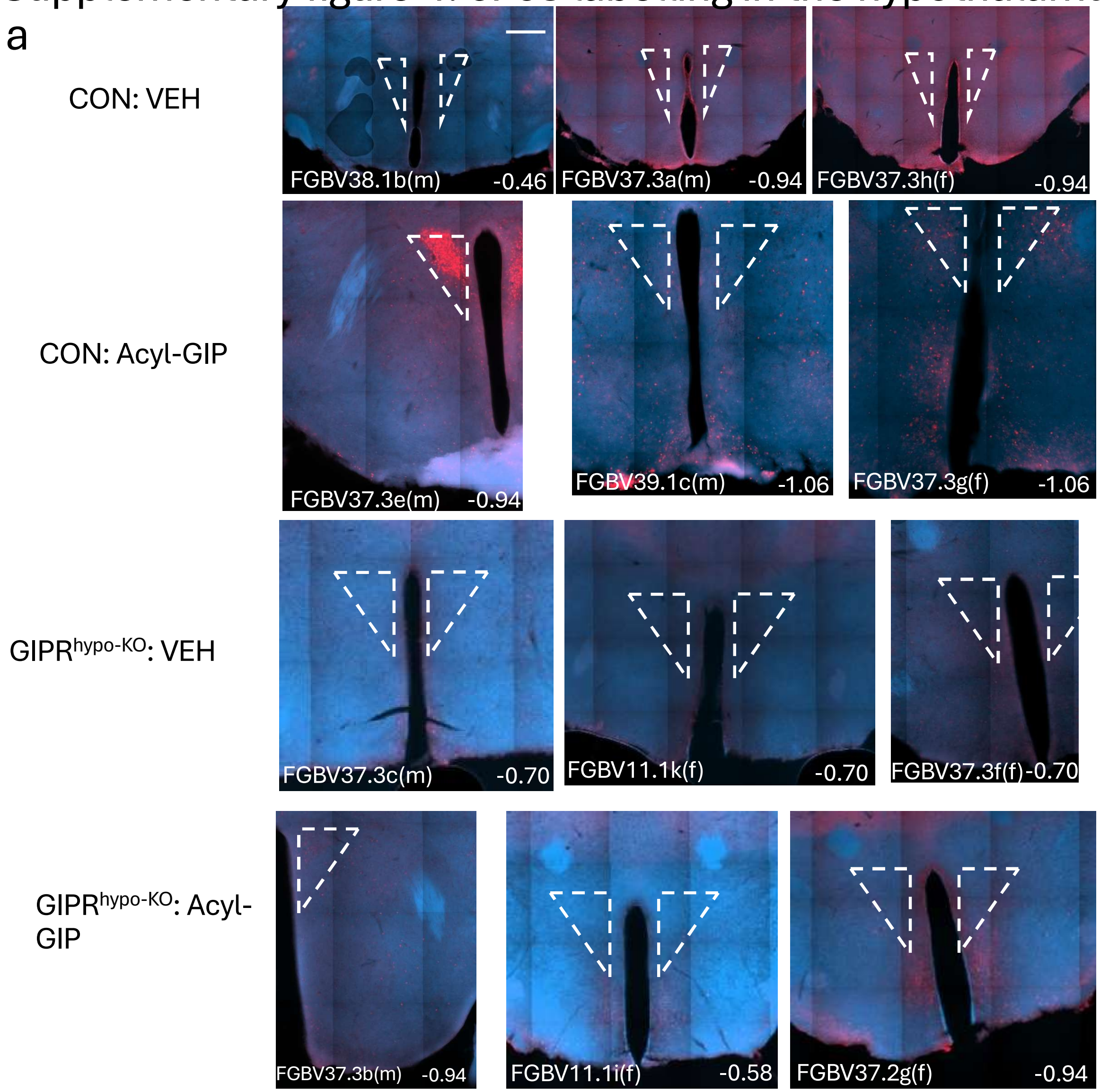

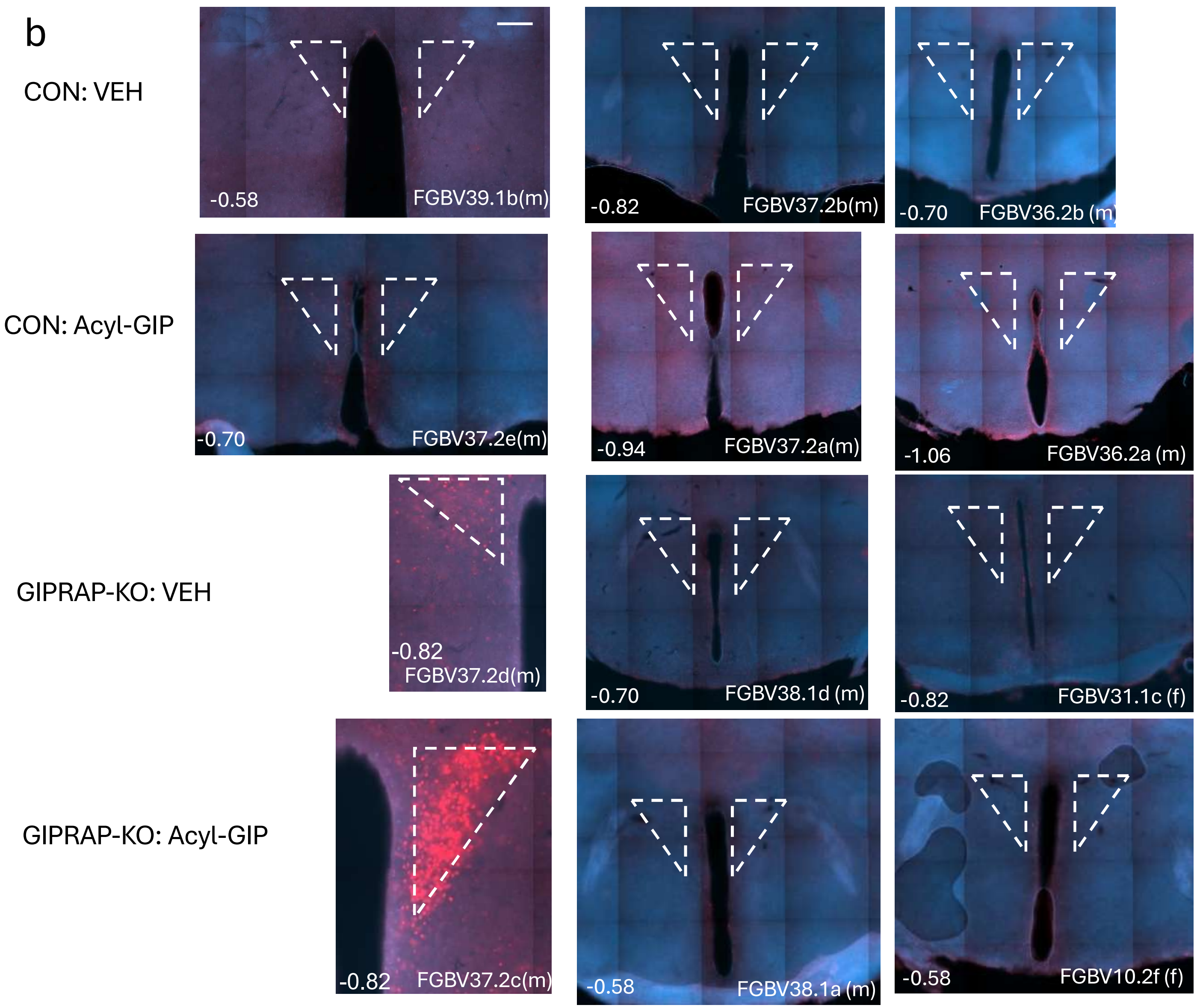

Supplementary figure 4: cFos labelling in the hypothalamus. Representative cfos images of the hypothalamus (PVN outlined, n =2-6 sections per mouse) of CON or a) Gipr$^{hypo\text{-}KO}$ or b) Gipr$^{AP\text{-}KO}$ mice receiving VEH or acyl-GIP (as indicated). FGBV denotes colony and animal ID (sex m/f as indicated). Bregma as specified, scale bar = 100µm.

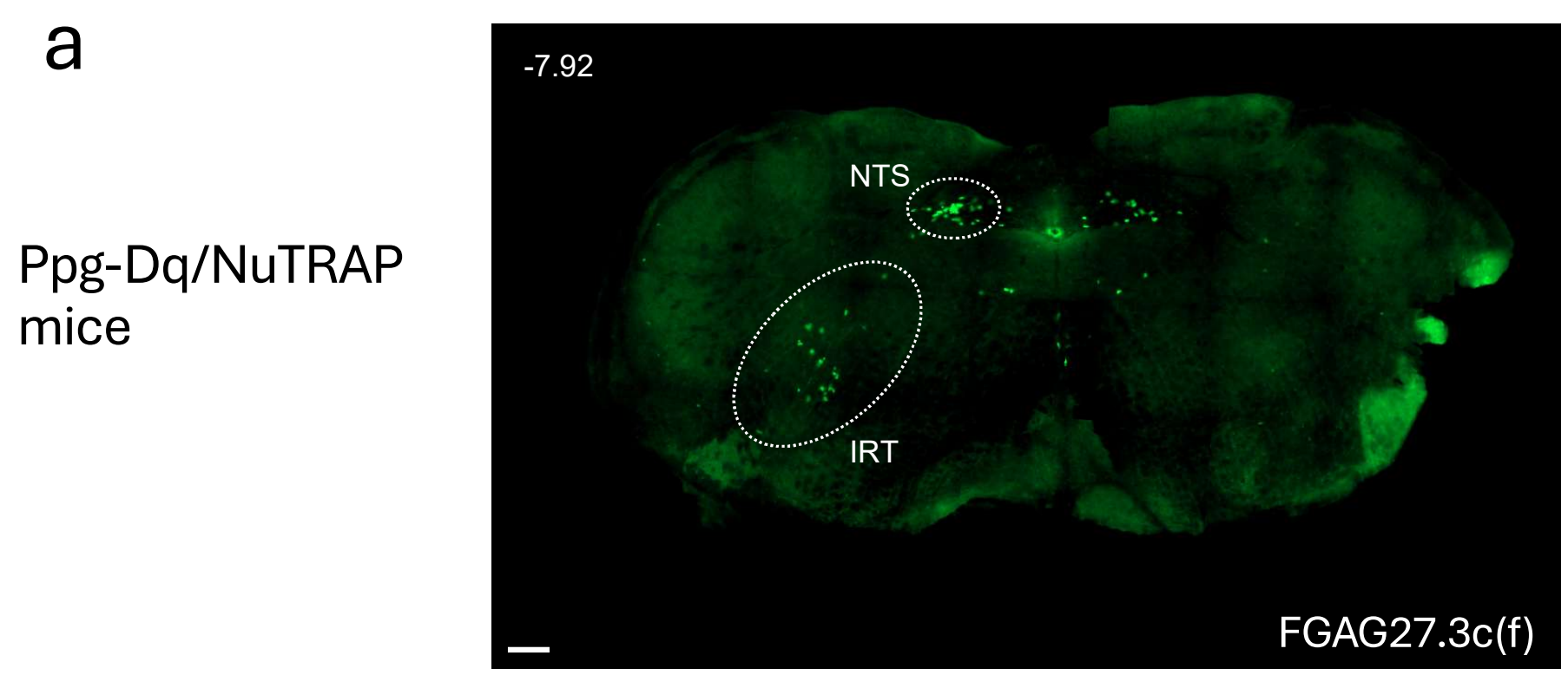

Supplementary figure 5: Targeting confirmation for PPG^{NTS} ablation. a) Representative image of fluorescently tagged PPG neurons in the brainstem. b) Images of brainstem PPG neurons following AAV-DTA to the NTS of Ppg-Dq mice (Ppg$^{NTS-Abl}$, n = 6); note reduced fluorescence in NTS but retained in IRT. FGAG denotes colony and animal ID (sex m/f as indicated). Bregma as specified, scale bar = 200μm.

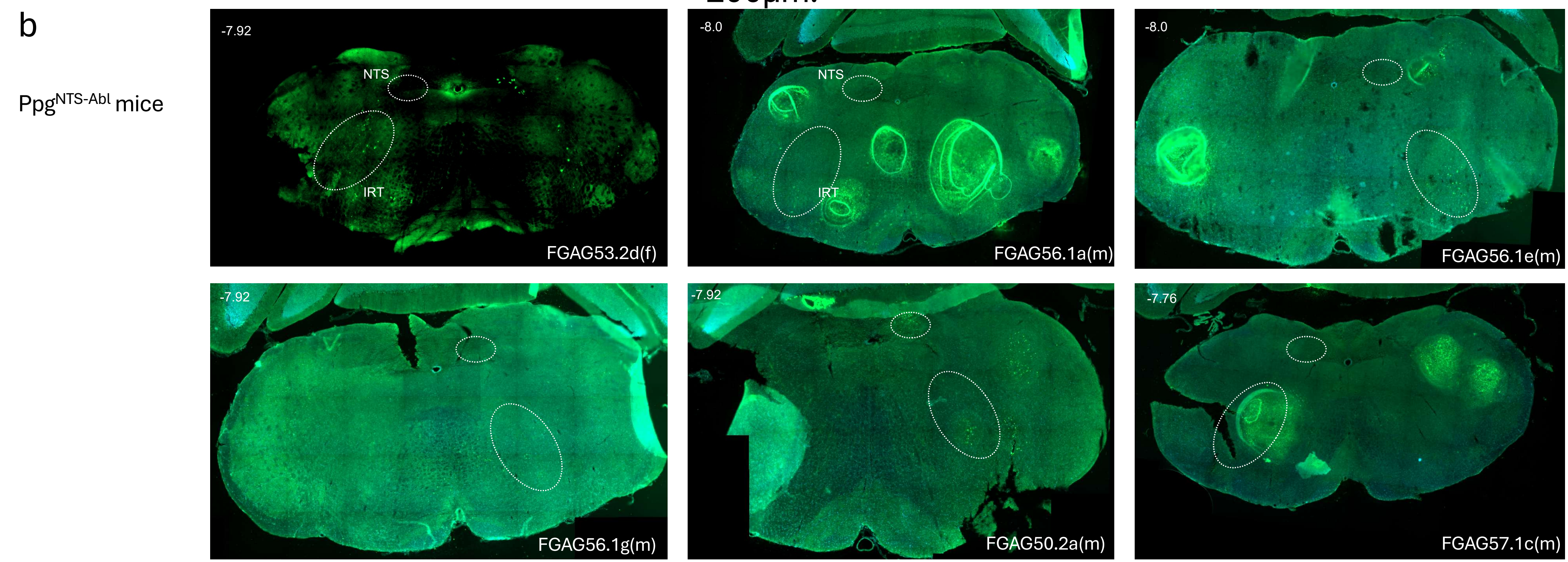

Supplementary Table 1: Antibody reagents

Antigen	Species	Concentration	Source	Secondary
Tdtomato	Rabbit	1:1000	Takara BioClontech 632,496	ThermoFisher Donkey anti-rabbit 555 (A31572)
Cfos	Rabbit	1:2000	SYSY 226008	ThermoFisher Goat anti-rabbit 555 (A21428)
GFP	Chicken	1:1000	Abcam 5450	ThermoFisher goat anti-chicken (A11039)
