# Supplementary material for: Distinct brain regions mediate regulation of food intake in response to GIPR agonism or antagonism
Source: Nat Metab. 2026 Jul 24;8(8):1669–78. doi: 10.1038/s42255-026-01575-z (PMC13489937; doi:10.1038/s42255-026-01575-z)
Supplement: Supplementary file 2 [file 42255_2026_1575_MOESM2_ESM.pdf]

nature portfolio

Corresponding author(s): Prof Frank Reimann, Fiona M Gribble

Last updated by author(s): Mar 23, 2026

Reporting Summary

Nature Portfolio wishes to improve the reproducibility of the work that we publish. This form provides structure for consistency and transparency in reporting. For further information on Nature Portfolio policies, see our Editorial Policies and the Editorial Policy Checklist.

Statistics

For all statistical analyses, confirm that the following items are present in the figure legend, table legend, main text, or Methods section.

n/a	Confirmed	
☐	☒	The exact sample size (n) for each experimental group/condition, given as a discrete number and unit of measurement
☐	☒	A statement on whether measurements were taken from distinct samples or whether the same sample was measured repeatedly
☐	☒	The statistical test(s) used AND whether they are one- or two-sided<br>*Only common tests should be described solely by name; describe more complex techniques in the Methods section.*
☒	☐	A description of all covariates tested
☐	☒	A description of any assumptions or corrections, such as tests of normality and adjustment for multiple comparisons
☐	☒	A full description of the statistical parameters including central tendency (e.g. means) or other basic estimates (e.g. regression coefficient) AND variation (e.g. standard deviation) or associated estimates of uncertainty (e.g. confidence intervals)
☐	☒	For null hypothesis testing, the test statistic (e.g. F, t, r) with confidence intervals, effect sizes, degrees of freedom and P value noted<br>*Give P values as exact values whenever suitable.*
☒	☐	For Bayesian analysis, information on the choice of priors and Markov chain Monte Carlo settings
☒	☐	For hierarchical and complex designs, identification of the appropriate level for tests and full reporting of outcomes
☒	☐	Estimates of effect sizes (e.g. Cohen's d, Pearson's r), indicating how they were calculated

Our web collection on statistics for biologists contains articles on many of the points above.

Software and code

Policy information about availability of computer code

Data collection	Zeiss Axioscan and sp8 associated software (Zen3 and Leica Application Suite X respectively) for image collection, subsequent viewing in ImageJ/Fiji.<br>Promethion metabolic cage software (Expedata version 1_9_27 or later)
Data analysis	Data were analysed using GraphPad Prism 9/10.<br>No code was used in the preparation of this manuscript.

For manuscripts utilizing custom algorithms or software that are central to the research but not yet described in published literature, software must be made available to editors and reviewers. We strongly encourage code deposition in a community repository (e.g. GitHub). See the Nature Portfolio guidelines for submitting code & software for further information.

Data

Policy information about availability of data

All manuscripts must include a data availability statement. This statement should provide the following information, where applicable:

- Accession codes, unique identifiers, or web links for publicly available datasets
- A description of any restrictions on data availability
- For clinical datasets or third party data, please ensure that the statement adheres to our policy

All data supporting the findings of this study are available within the paper

Research involving human participants, their data, or biological material

Policy information about studies with human participants or human data. See also policy information about sex, gender (identity/presentation), and sexual orientation and race, ethnicity and racism.

Reporting on sex and gender	*Use the terms sex (biological attribute) and gender (shaped by social and cultural circumstances) carefully in order to avoid confusing both terms. Indicate if findings apply to only one sex or gender; describe whether sex and gender were considered in study design; whether sex and/or gender was determined based on self-reporting or assigned and methods used. Provide in the source data disaggregated sex and gender data, where this information has been collected, and if consent has been obtained for sharing of individual-level data; provide overall numbers in this Reporting Summary. Please state if this information has not been collected. Report sex- and gender-based analyses where performed, justify reasons for lack of sex- and gender-based analysis.*
Reporting on race, ethnicity, or other socially relevant groupings	*Please specify the socially constructed or socially relevant categorization variable(s) used in your manuscript and explain why they were used. Please note that such variables should not be used as proxies for other socially constructed/relevant variables (for example, race or ethnicity should not be used as a proxy for socioeconomic status). Provide clear definitions of the relevant terms used, how they were provided (by the participants/respondents, the researchers, or third parties), and the method(s) used to classify people into the different categories (e.g. self-report, census or administrative data, social media data, etc.) Please provide details about how you controlled for confounding variables in your analyses.*
Population characteristics	*Describe the covariate-relevant population characteristics of the human research participants (e.g. age, genotypic information, past and current diagnosis and treatment categories). If you filled out the behavioural & social sciences study design questions and have nothing to add here, write "See above."*
Recruitment	*Describe how participants were recruited. Outline any potential self-selection bias or other biases that may be present and how these are likely to impact results.*
Ethics oversight	*Identify the organization(s) that approved the study protocol.*

Note that full information on the approval of the study protocol must also be provided in the manuscript.

Field-specific reporting

Please select the one below that is the best fit for your research. If you are not sure, read the appropriate sections before making your selection.

☒ Life sciences ☐ Behavioural & social sciences ☐ Ecological, evolutionary & environmental sciences

For a reference copy of the document with all sections, see nature.com/documents/nr-reporting-summary-flat.pdf

Life sciences study design

All studies must disclose on these points even when the disclosure is negative.

Sample size	Sample size was determined from the existing literature and pilot studies within the author's laboratory.
Data exclusions	Exclusion criteria were pre-established. Tissue sections from mice involved in metabolic phenotyping were prepared (as described) and fluroscent reporter expression verified. Any mice that exhibited inaccurate viral targeting or transduction efficiency were excluded from the anaylysis, which accounted for less than 10% of mice across the experiments.
Replication	The experiments evaluating the impact of pharmacological treatment were performed in multiple cohorts - these were subsequently combined. The manuscript's findings are supported by converging evidence, ensuring the strength and reproducibility of the conclusions.
Randomization	Animals were semi-randomized to treatment groups to ensure balance in terms of age, weight and sex. Where possible the experimentators were blinded to group allocation during data collection and analysis.
Blinding	During the data collection phase of in vivo experiments, the investigators were blinded to both the virus and drug treatment across groups (where possible).

Reporting for specific materials, systems and methods

We require information from authors about some types of materials, experimental systems and methods used in many studies. Here, indicate whether each material, system or method listed is relevant to your study. If you are not sure if a list item applies to your research, read the appropriate section before selecting a response.

Materials & experimental systems

n/a	Involved in the study
☐	☒ Antibodies
☒	☐ Eukaryotic cell lines
☒	☐ Palaeontology and archaeology
☐	☒ Animals and other organisms
☒	☐ Clinical data
☒	☐ Dual use research of concern
☒	☐ Plants

Methods

n/a	Involved in the study
☒	☐ ChIP-seq
☒	☐ Flow cytometry
☒	☐ MRI-based neuroimaging

Antibodies

Antibodies used

These are provided in the paper and reproduced below:

Tdtomato Rabbit 1:1000 Takara BioClontech 632,496 ThermoFisher Donkey anti-rabbit 555 (A31572)
Cfos Rabbit 1:2000 SYSY 226008 ThermoFisher Goat anti-rabbit 555 (A21428)
GFP Chicken 1:1000 Abcam 5450 ThermoFisher goat anti-chicken (A11039)

Validation

The primary and secondary antibodes listed have been widely employed in mouse models for the purposes of IHC as per this stidy. Detailed information is accessible via the companies' websites

Animals and other research organisms

Policy information about studies involving animals; ARRIVE guidelines recommended for reporting animal research, and Sex and Gender in Research

Laboratory animals

All experiments were performed in adult (>6 weeks) male and female mice, on a C57BL/6 background. The transgenetic colony of GIPRflx/flx, GIPR-Cre and PPG-Cre mice are maintained at the University of Cambridge. All mice were fed a std lab chow, unless otherwise stated. Chronic HFD studies, performed to induce obesity, were conducted in male mice only. This is stated in the methods.

Wild animals

No wild animals were used in this study.

Reporting on sex

The results were obtained in male and female mice, as obesity affects both men and women, and weight loss drugs are used by both sexes. Chronic HFD studies, performed to induce obesity, were limited to male mice (as discussed). Treatment groups were balanced for sex.

Field-collected samples

No field-collected samples were used.

Ethics oversight

All experiments were performed under the UK Home Office project licence PE50F6065 in accordance with the UK Animals (Scientific Procedures) Act, 1986 and approved by the University of Cambridge Animal Welfare and Ethical Review Body.

Note that full information on the approval of the study protocol must also be provided in the manuscript.

Plants

Seed stocks

Report on the source of all seed stocks or other plant material used. If applicable, state the seed stock centre and catalogue number. If plant specimens were collected from the field, describe the collection location, date and sampling procedures.

Novel plant genotypes

Describe the methods by which all novel plant genotypes were produced. This includes those generated by transgenic approaches, gene editing, chemical/radiation-based mutagenesis and hybridization. For transgenic lines, describe the transformation method, the number of independent lines analyzed and the generation upon which experiments were performed. For gene-edited lines, describe the editor used, the endogenous sequence targeted for editing, the targeting guide RNA sequence (if applicable) and how the editor was applied.

Authentication

Describe any authentication procedures for each seed stock used or novel genotype generated. Describe any experiments used to assess the effect of a mutation and, where applicable, how potential secondary effects (e.g. second site T-DNA insertions, mosiacism, off-target gene editing) were examined.
