# Supplementary material for: Distinct brain regions mediate regulation of food intake in response to GIPR agonism or antagonism
Source: Nat Metab. 2026 Jul 24;8(8):1669–78. doi: 10.1038/s42255-026-01575-z (PMC13489937; doi:10.1038/s42255-026-01575-z)
Supplement: Supplementary file 3 [file 42255_2026_1575_MOESM3_ESM.pdf]

nature portfolio

Peer Review File

Distinct brain regions mediate regulation of food intake in response to GIPR agonism or antagonism

Corresponding Author: Professor Fiona Gribble

Version 0:

Reviewer comments:

Reviewer #1

(Remarks to the Author)
In the presented work, Lewis and colleagues report that agonism and antagonism of the GIP receptor regulate food intake and body weight via distinct areas in the brain (namely the hypothalamus and the hindbrain), thereby significantly contributing to finally solving the conundrum as to why, where and how GIPR (ant)agonism accelerates GLP-1-induced weight loss in obese rodents. In a series of elegantly designed and well conducted studies, the authors assessed the metabolic effects of GIPR agonism or antagonism in adjunct to GLP-1R agonism in mice with targeted deletion of GIPR in either the area postrema (AP) or hypothalamus. Significantly expanding current knowledge on the role of GIPR signal modification in regulation of systems metabolism, the authors convincingly demonstrate that body weight loss and suppression of food intake induced by GIPR agonism, but not antagonism, originates from GIPR signal modification in the area postrema (and is hence compromised in mice with AP-specific deletion of GIPR), while that of GIPR antagonism (but not agonism) originates from GIPR signal modification in the hypothalamus. The authors hence not only confirm previous data indicating that GIPR agonism and antagonism decrease body weight and food intake via different mechanisms but also show that this originates from GIPR signal modification in distinct brain areas controlling energy metabolism.

The manuscript is very well written, easy to follow, and of substantial importance for the field. It adds significant value to finally understand how drugs stimulating or inhibiting the central GIP system affect energy metabolism, thereby paving the way to specifically target the GIP system using next generation drugs. The study is without question of high scientific value. The target journal is considered adequate for these important and significant data. The group is well established at the forefront of GIP biology, and the reported data are solid and sound. I have only a set of rather minor comments and suggestions that the authors may find useful.

In the abstract, the authors mention that GIPR agonism decreases body weight via GABAergic GIPR neurons. Here I suggest to also cite the most recent paper from Randy Seeley, confirming the initial report published in Nat Metab (PMID: 39612941). Furthermore, when saying "…also likely through a central effect, as similar observations were made in global KO mice or mice lacking the Gipr in Nestin-expressing cells (3, 8, 9)", I suggest to also mention that also mice with lack of GIPR in GABAergic neurons show decreased body weight relative to WT controls (PMID: 39612941, PMID: 37946085).

Fig 1b and EDF1a, it would be nice to show WT and KO mice side by side to allow for the accurate deletion of GIPR in the AP. Or is the first panel of EDF1a showing a WT mouse and the others a KO mouse? Clearly labelling the panels with WT and KO would help here to increase readability of this Figure.

Figure 1c-e. Showing a bar graph for the body weight development in chow fed mice is a bit suboptimal. A longitudinal graph with body weight development over time would have been more optimal. Also, I don't understand what the black symbols within the bar graphs are showing here. The orange circles obviously correspond to the KO individuals, but the same bar graph also contains black symbols. Maybe good to clarify this, especially since these symbols appear in Fig 1C-E but not I and J.

Fig 1l. The lower food intake in the AP-GIPR KO is quite striking, with the KO mice eating less than half compared to the WT. This degree of food intake suppression would notably be expected to result in a much greater decrease in body weight relative to what is shown in Fig 1h. While being largely consistent with the body weight phenotype, this indicates that food intake changes are best measured over several days. Food intake shows quite some changes from day to day, and measurement over several days would have given a more accurate measure on the genotype related differences in food intake (just saying). While this statement is obviously of minor relevance in case of a positive finding, it may become in issue when data are less obvious.

Fig 1m. Although the data are speaking a clear language, I suggest using a higher magnification to peal out the beauty of these data.

Fig 1q. These are beautiful measures. I suggest adding here a sentence, saying that these data further support the notion that acyl-GIP acts as a GIPR agonist but not as a GIPR antagonist. While maybe sounding redundant, it serves the purpose of again making clear that GIPR agonism does not lead here to GIPR antagonism (which is by some people still suggested to be a reasonable cause of GIPR agonism induced weight loss). On the same heels, I suggest changing the following sentence "These data provide high resolution validation that GIP directly activates AP neurons" into "These data provide high resolution validation that GIP directly activates but not inactivates AP GIPR neurons" .

EDF2. I appreciate the reproducibility of acyl-GIP to decrease body weight and food intake. While also these data are solid and sound, I would have preferred seeing such data in a more longitudinal fashion, i.e. with daily administration and metabolic effects on body weight and food intake over several days. Since every paper stands on its own, demonstration of drug effects over several days adds a high level of sophistication that goes beyond acute drug effects. In other words, acute drug effects are nice, more chronic drug effects are better . But this does notably not compromise the beauty and relevance of the here shown data.

Fig2i and j. The suppression of food intake induced by acyl-GIP is quite dramatic in this study. It would be good to indicate which dose was used here. I can't find this in the text or the figure legends. How does this dose compare to the used dose of acyl-GIP in previous manuscripts? I suggest including doses in every figure legend. Did you used here 100 nmol/kg, as indicated for the CTA test? I also noted that some panels here comprise only n=3-5 mice. I personally think that in vivo data on food intake that are obtained in as little as n=3 mice are quite suboptimal. Nonetheless, the data are consistent with what has been previously reported for acyl-GIP in mice with germline GABAergic GIPR KO. So, despite being seemingly of limited statistical power, the data are legitimate.

EDF3g,h. I encourage the authors to blot individual data on energy expenditure against body weight, followed by ANCOVA analysis using body weight as co-variate (PMID: 40993210). It has the advantage that changes in expenditure are then given in relation to body weight, which is important especially when using drugs that decrease body weight.

When saying "Thus, although GIPR in the AP are required for the weight loss effects of GIPR agonists, they are not the target of therapeutic GIPR-antagonists, supporting recent findings that Gipr KO in GABAergic neurons also abolished responses to GIPR agonism but not antagonism.", I suggest to also add the respective reference here (PMID: 40360757).

Fig 4a. Apart from confirming successful targeting of the hypothalamus, it would be nice seeing successful target gene silencing. This can obviously be done e.g. using RNAscope, which the authors have been used in previous publications to demonstrate successful GIPR KO (but admittedly, successful target gene silencing is unquestioned given the nice functional read outs in Fig 4h-k).

The results presented in Fig 3 and 4 are very beautiful and make unmistakably clear that GIPR agonism and antagonism decrease body weight and food intake via different brain areas. However, one important point I want to make here is that acyl-GIP equally decreases body weight in DIO WT and GLP-1R KO mice (PMID: 33571454). To me, it's now becoming increasingly clear that GIPR antagonism, but not agonism, decreases body weight by accelerating GLP-1-induced weight loss. In case the authors are up for walking a few additional miles, it would be very interesting to study the co-therapy of Lira + GIPR antagonist in DIO mice with hypothalamic- and AP-specific lack of GLP-1R. If the aforementioned hypothesis is correct, then peripheral administration of the Lira + GIPR antagonist co-therapy should not decrease body weight and food intake in mice with hypo GLP-1R Ko, but preserved weight loss on mice with AP-GLP-1R ko. Such studies would add significantly to mechanisms of how GIPR antagonism decreases body weight. But I would understand if the authors prefer to rather address this then in the next paper.

Reviewer #2

(Remarks to the Author)
The manuscript by Lewis et al. explores the neural underpinnings of the paradoxical weight-reducing effects of both GIPR agonism and antagonism, identifying distinct roles for GIPR populations in the area postrema and hypothalamus. The question is timely and conceptually well framed, and the text flows logically with clear interpretation and alignment to the broader incretin literature. However, the quantitative foundation of several key claims is less convincing. Group sizes in pivotal in vivo experiments are small, and the statistical treatment often appears ad hoc, for example, it is unclear what justifies the few comparisons presented in Fig. 4N across eight groups. Consequently, results that should consolidate the field instead leave the reader uncertain, despite data and text suggesting a clear directionality. The graphical presentation is also highly inconsistent, with variable line thickness, uneven plotting of individual data points, and omission of correct time axes in glucose tolerance tests, giving the impression of figures assembled hastily. Overall, the narrative is stronger than the evidence that supports it, and the study would benefit substantially from more rigorous statistical analysis, replication, and harmonized figure design.

Version 1:

Reviewer comments:

Reviewer #1

(Remarks to the Author)
I think that the manuscript has strenghtened quite a bit and consider the data ready for publication.

The manuscript deserves publication. But I also want to point out that the authors are not striving, in my opinion, for the highest scientific standards. For example, measuring food intake longitudinally can be done quite easily simply by dividing the cage food consumption by the number of mice in the cage. Moreover, I am concerned about the authors tendency to continuously use very low sample sizes. Even the now used n=5-6 mice per group is very suboptimal – n=8 is typically considered standard in the field. I am worried that studies like this one here might be used as an example to define a new (lower) scientific standard and that ethical committees – perhaps first in the UK but then, eventually, in other countries as well - will no longer allow performing rigorous experiments using higher sample sizes. I consider it also a responsibility of the journal to maintain high standards to ensure rigor and a high level of reproducibility.

Reviewer #2

(Remarks to the Author)
I have now had a chance to go through the revised manuscript and I think the authors have appropriately addressed most of my main concerns, so happy to recommend publication.

Open Access This Peer Review File is licensed under a Creative Commons Attribution 4.0 International License, which permits use, sharing, adaptation, distribution and reproduction in any medium or format, as long as you give appropriate credit to the original author(s) and the source, provide a link to the Creative Commons license, and indicate if changes were made.
In cases where reviewers are anonymous, credit should be given to 'Anonymous Referee' and the source.
The images or other third party material in this Peer Review File are included in the article's Creative Commons license, unless indicated otherwise in a credit line to the material. If material is not included in the article's Creative Commons license and your intended use is not permitted by statutory regulation or exceeds the permitted use, you will need to obtain permission directly from the copyright holder.
To view a copy of this license, visit https://creativecommons.org/licenses/by/4.0/
